# Enhancing the miRNA Detection Sensitivity of DNA Origami Book Biosensors Using Lock Modifications and a Polymer Additive

**DOI:** 10.1002/smll.202512100

**Published:** 2025-11-14

**Authors:** Ivana Domljanovic, Samet Kocabey, Guillermo P. Acuna, Curzio Ruegg

**Affiliations:** ^1^ Laboratory of Experimental and Translational Oncology Department of Oncology Microbiology and Immunology Faculty of Science and Medicine University of Fribourg Chemin du Musée 18, PER17 Fribourg 1700 Switzerland; ^2^ NCCR Bio‐inspired Materials University of Fribourg Fribourg 1700 Switzerland; ^3^ Photonic Nanosystems Department of Physics Faculty of Science and Medicine University of Fribourg Chemin du Musée 3, PER08 Fribourg 1700 Switzerland; ^4^ XEMPERIA SA Marly Innovation Center Route de l'Ancienne Papeterie 180 Marly 1723 Switzerland

**Keywords:** breast cancer, DNA origami biosensor, FRET, miRNA, quenching

## Abstract

DNA origami‐based biosensors provide a powerful and programmable platform for sensitive and specific molecular diagnostics. In this study, a dynamic DNA origami book biosensor is validated for direct detection of microRNA21 (miR‐21) in complex biological fluids, including human serum and plasma. Two optical readouts are engineered and tested: Förster resonance energy transfer (FRET) and fluorescence quenching using precisely arranged donor‐acceptor fluorophore pairs. To improve sensitivity, detection occurs under varied conditions, including modifications to the lock mechanism (fluorine‐modified bases and locked nucleic acid) and the addition of a biocompatible polymer, diethylene glycol (DEG). The biosensor maintains specificity in spiked serum, plasma, and clinical plasma samples from breast cancer patients. Single origami nanostructure detection experiments show that target miRNAs are identified within 10 min, with limits of detection (LoD) of 0.69 pm in buffer with LNA, 8.86 pm in 100% serum, and 23.24 pm in 100% plasma, without enzymatic amplification. Importantly, simultaneous detection of miR‐21 and miR‐7a in clinical plasma samples is demonstrated, underscoring the platform's potential for personalized diagnostics and liquid biopsy applications. These results show that tuning the lock mechanism and adding polymers enhances sensitivity and enables adaptable performance, advancing nanotechnology‐based biomarker profiling, including diverse microRNA detection.

## Graphical abstract

A dynamic DNA origami book biosensor enables simultaneous detection of microRNA‐21 (miR‐21) and microRNA‐7a (miR‐7a) in plasma from breast cancer patients. Using fluorescence resonance energy transfer (FRET) and quenching readouts, this programmable nanostructure achieves enzyme‐free detection within 10 minutes, highlighting its potential for sensitive, specific, and multiplex molecular diagnostics in personalized cancer biomarker profiling.

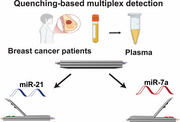

## 1 Introduction

Circulating microRNAs (miRNAs) have emerged as a central focus of biomedical research over the past decade due to their vital roles as regulators of gene expression and their significant association with various disease states, including cancer, cardiovascular conditions, neurodegenerative diseases, and inflammatory disorders.^[^ ^1^, ^2^, ^3^, ^4^, ^5^ ^]^ Recent advances have reinforced the notion that distinct miRNA profiles found in blood and other biofluids may potentially indicate early‐stage tumor development, response to treatment, and even minimal residual disease well before clinical symptoms appear.^[^ ^2^, ^6^, ^7^, ^8^ ^]^ This has made miRNAs highly attractive as biomarkers for non‐invasive early detection, prognosis, and monitoring of various cancers.^[^ ^4^, ^9^, ^10^ ^]^

Despite their promise and potential use in clinical medicine, reliable detection of miRNAs in the blood remains technically challenging. The fact that circulating miRNAs are typically present at low concentrations (low picomolar levels to femtomolar) and often exhibit high sequence homology between family members, sometimes differing by only a single nucleotide, complicates their specific and sensitive detection.^[^ ^11^, ^12^ ^]^ Traditional detection methods, such as quantitative PCR (qRT‐PCR), next‐generation sequencing (NGS), and microarrays, have good performance profiles. However, they require specific competencies, are time‐consuming and expensive, thereby limiting their widespread clinical use.^[^ ^13^ ^]^ Moreover, these techniques have limitations, making accurate (single‐nucleotide) discrimination between similar miRNAs difficult due to cross‐hybridization or sequencing errors. They also often require extensive post‐processing steps.^[^ ^13^ ^]^

The pressing demand for straightforward, swift, highly targeted, and multiplex miRNA detection technologies, especially those that function directly in crude biological fluids such as serum and plasma, has driven innovation at the crossroads of nanotechnology, biosensing, and molecular diagnostics.^[^ ^14^, ^15^ ^]^ Recent clinical efforts, such as extensive biomarker discovery groups and early cancer detection initiatives (including the Galleri trial and the EU Cancer Mission), highlight the pivotal importance of liquid biopsies and circulating small RNAs for the future of precision medicine.^[^ ^16^ ^]^

DNA nanotechnology, especially the DNA origami technique, offers a robust framework for addressing these challenges. DNA origami enables the construction of dynamic nanoscale structures with precisely positioned functional groups, capable of undergoing conformational changes in response to specific molecular interactions.^[^ ^17^, ^18^, ^19^ ^]^ Fluorophore networks engineered onto these structures can produce strong optical signals through Förster resonance energy transfer (FRET) or quenching effects, enabling highly sensitive and specific detection at the single‐molecule level without the need for enzymatic target amplification.^[^ ^19^, ^20^, ^21^ ^]^

In a previous study, we introduced a dynamic DNA origami book biosensor platform specifically designed to detect multiple cancer‐related miRNAs simultaneously.^[^ ^19^ ^]^ Unlike conventional biosensors, this platform features two optical readout methods: a FRET‐based detection mode for one miRNA target and a quenching‐based mode for two miRNA targets, based on arrays of fluorophore‐quenchers or donor‐acceptor pairs decorated on the book structure. Additionally, the modular lock system of the biosensor enables straightforward adaptation to various miRNA targets, facilitating multiplexing within a single assay. Using this biosensor, we demonstrated that it could detect target nucleic acids at concentrations as low as 1–10 pm within 10 min after target exposure.

In this study, we validated the book biosensor for detecting clinically relevant miRNAs, particularly miR‐21, miR‐7a, in complex fluids, including human serum and plasma, and breast cancer patient plasma samples. We further enhanced the stability of the structures by applying chemical modifications (2′‐fluoro (2′‐F) and lock nucleic acids LNA) to the locks that bind to target miRNAs, and increased sensitivity by utilizing a biocompatible polymer additive (diethylene glycol). The platform demonstrated low picomolar sensitivity, specificity, and potential for multiplexed detection, supporting its translation toward laboratory research‐based technology and clinical diagnostics.

## 2 Results and Discussion

### 2.1 Single‐Origami Analysis of DNA Origami Book Biosensors using Wide‐Field Fluorescence Microscopy Across Diverse Biological Environments

**Figure 1:**
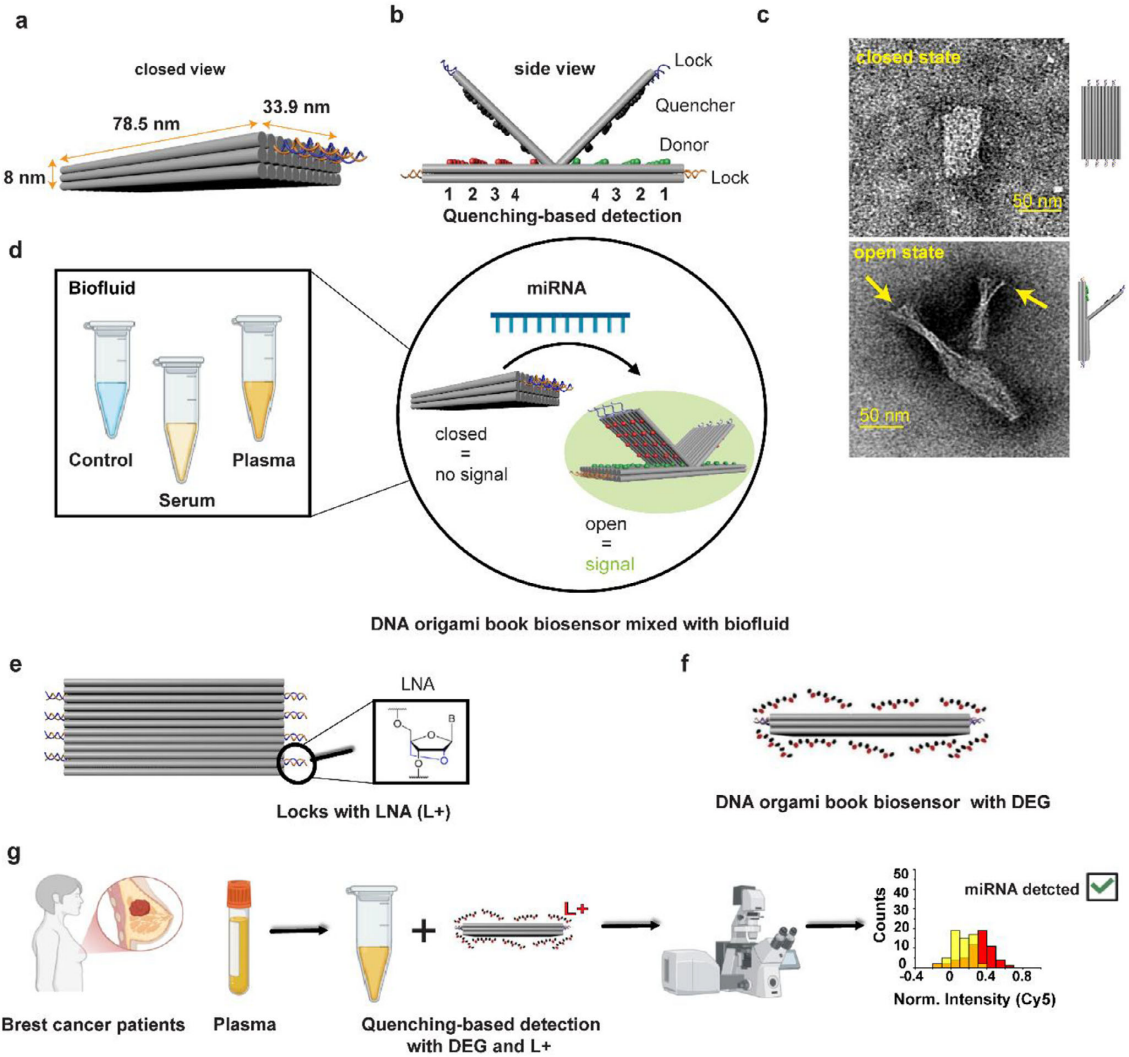
Design, functional states, and fluid compatibility of a DNA origami book biosensor platform. a) A 3D schematic illustrates the DNA origami biosensor in its closed book conformation, held together by two locks (L). Image taken from a previous publication.^[^ ^19^ ^]^ b) Green, red, and black spheres represent the arrays of donor and quencher groups. In total, 20 fluorophore/quencher pairs were positioned on each side. They are divided into columns with 1 column (5 donors or quenchers), 2 columns (10 of them), and 4 columns (20 of them). When open, representing the detection of miRNA, the lock strands are released, allowing the donor and quencher fluorophores to separate, enabling the observation of the difference in fluorescence signaling. Image taken from a previous publication.^[^ ^19^ ^]^ c) A transmission electron microscopy (TEM) image shows the DNA origami biosensor in both open and closed states during imaging. Image taken from a previous publication.^[^ ^19^ ^]^ d) The biosensor's structural response when incubated in various biological fluids that are spiked with synthetic miRNA, the target of our interest: control (buffer), serum, and plasma. e) The DNA origami biosensor with locks modified with LNA(L+). f) The closed‐state DNA origami biosensor mixed with DEG. g) Plasma from a breast cancer patient was incubated with DNA origami biosensors containing LNA‐modified locks. To enhance assay sensitivity, DEG was added. Fluorescence changes were visualized using wide‐field microscopy, and the resulting increase in fluorescence intensity is represented in the histogram.

To assess the performance of our DNA origami book biosensor under biologically relevant conditions, we conducted a systematic evaluation across a range of environments. The design, functional states, and testing conditions of the DNA origami book biosensor are given in **Figure** **1**. Initial testing was performed in buffer to establish baseline performance, followed by experiments in human serum and plasma at increasing concentrations (30%, 50%, and 100%) to replicate the complexity of physiological media. As a model analyte, we selected miR‐21, a clinically significant biomarker highly expressed in various cancer types.^[^ ^22^, ^23^, ^24^ ^]^ Its low GC content (36.4%) is known to reduce hybridization efficiency and increase the difficulty of detection.^[^ ^25^ ^]^ Testing a low‐GC‐content sequence allowed us to assess the biosensor's sensitivity under challenging conditions. To quantify biosensor response and determine the limit of detection (LoD), synthetic miR‐21 was spiked into each biofluid condition. After establishing baseline detection, we introduced chemical modifications to the locking strands, specifically locked nucleic acids (LNA) and 2′‐fluoro (2′‐F) analogs, to improve probe stability and hybridization affinity. Additionally, diethylene glycol (DEG) was incorporated in solution as a polymer additive to modulate hybridization kinetics and reduce nonspecific interactions, thereby enhancing both sensitivity and structural stability. This stepwise evaluation allowed for a comprehensive assessment of the biosensor's robustness and diagnostic potential across increasingly challenging environments.

### 2.2 Performance of the Book Biosensor in Control Conditions

**Figure 2:**
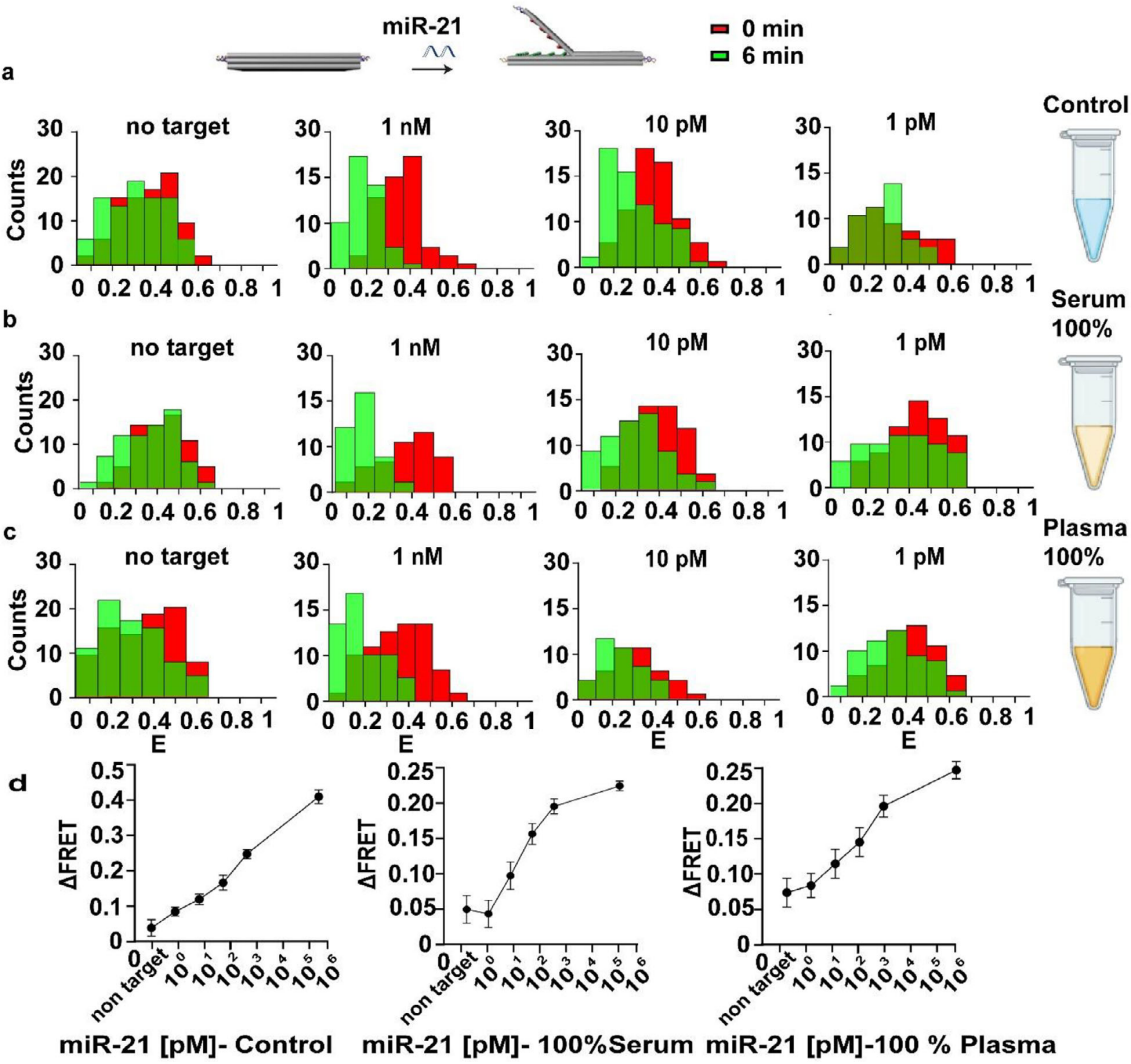
FRET‐based detection of miR‐21 using DNA origami book biosensors across different biofluids. a) Representative histograms of FRET efficiency distributions for DNA origami book biosensors incubated in control (buffer 1x TAE 12 mm Mg^2+^) after exposure to different concentrations of synthetic miR‐21 (1 nm, 10 pm, 1 pm), and in the presence of a non‐target miRNA (miR‐342) serving as a specificity control (“non‐target”). Histograms show the distribution of biosensors in closed (high‐FRET, red, 0 min) and open (low‐FRET, green, 6 min) states, indicating a conformational change upon target recognition. b) Identical experimental conditions were applied in 100% human serum. c) Identical experimental conditions were applied 100% human plasma. d) Summary plot showing dose‐dependent ΔFRET response in control, 100% serum, and 100% plasma, analyzed using log‐linear regression over the dynamic range of measured concentrations. Linear regression was then applied to the lower concentration range. Note: E represents FRET efficiency, and count is the number of DNA origami biosensors observed.

To begin, we conducted a control test to evaluate the miRNA detection capabilities of our book biosensor using synthetic miR‐21 spiked in control (buffer 1x TAE 12 mm Mg^2+^). The book biosensors were assembled with columns (1–3) of Cy3 and Cy5, followed by purification, and then immobilized onto glass slides by biotin‐streptavidin. Images were then captured every 2 min for 10 min, with the target introduced after 1 min. The intensity from the DNA origami biosensor structures was extracted, and the FRET efficiency was calculated. Additionally, we imaged the structures before introducing the target in the same chamber, but at different locations, to assess the laser's impact on the fluorophores and monitor the structures' behavior, confirming minimal photobleaching. Our findings indicated that adding non‐target miRNA caused only a slight change in FRET efficiency (5%). In contrast, introducing the synthetic miR‐21, the target of our interest, produced a significant average FRET efficiency change from 41% to 12% for concentrations ranging from 1 µm to 10 pm. The lowest concentration of miR‐21 added was 1 m, and the change in FRET was ≈8% (**Figure** **2**a). We estimated the theoretical LoD of miR‐21 based on the measured normalized counts of the non‐target miRNA. The change in FRET response was first analyzed using log‐linear regression over the dynamic range of measured concentrations. A linear regression was then applied to the lower concentration range, near the baseline response, to calculate LoD, which was determined to be 4.5 pM (Figure 2d). The main results are represented in Figure 2a, and the corresponding histograms for the remaining results are provided in Figure S1a (Supporting Information).

In a previous study using the same book biosensor platform, we reported miRNA detection using miRNA342, a target with significantly higher GC content (≈55%), which resulted in a lower LoD.^[^ ^19^ ^]^ The current performance with miR‐21 demonstrates that targets with low GC content yield higher detection thresholds. This is consistent with the literature showing that low‐GC sequences form less stable duplexes, leading to weaker binding affinity and elevated LoDs.^[^ ^26^, ^27^ ^]^ For instance, profiling platforms have shown that miRNAs with less than 35% GC are among the least efficiently detected through sequencing and hybridization.^[^ ^25^ ^]^ In contrast, probes targeting high‐GC sequences benefit from increased hydrogen bonding and stacking interactions, resulting in a stronger and more stable hybridization and greater assay sensitivity. Our results highlight the sequence‐dependent limitations of nucleic acid‐based biosensors.

### 2.3 Performance of the Book Biosensor in Serum and Plasma

Next, we studied the behavior of the book biosensor in complex environments, particularly in healthy human serum at 3 different concentrations: 30%, 50%, and 100%. We assembled the same DNA origami biosensor used in the control study and experimented as described above. Minimal photobleaching from laser exposure was observed, confirming that the FRET changes were predominantly target‐induced. In all tested serum conditions, adding 1 µm non‐target RNA induced minor changes in FRET efficiency (6% in 30% serum, 4% in 50% serum, and 5% in 100% serum), demonstrating high specificity (Figure 2b; Figure S1b,c, Supporting Information). Upon addition of the synthetic miR‐21 target, we observed significant, concentration‐dependent changes in FRET efficiency. In 30% serum, the FRET response exhibited concentration‐dependent sensitivity, with FRET changes of 24%, 8.5%, and 5% observed at target concentrations of 1 µm, 10 pm, and 1 pm, respectively (Figure S1b, Supporting Information). Calculate LoD was determined to be 6.87 pm.

A similar concentration‐dependent response was observed in 50% serum, with FRET changes of 23% at 1 µm, 8.6% at 10 pm, and 4% at 1 pm, resulting in an estimated LoD of 7.94  pm (Figure S1c, Supporting Information). In 100% serum, the platform showed a FRET efficiency change of 23% at 1 µm, 9.1% at 10 pm, and 3.6% at 1 pm (Figure 2b). The calculated LoD was found to be 8.86 pm (Figure 2d). The remaining data for fluorescence histograms are included in Figure S1b,c,d,h (Supporting Information).

To further assess the performance of our book biosensors in biofluids, we conducted experiments in human plasma at 3 concentrations: 30%, 50%, and 100%. The experiment followed the same procedures as the control and serum studies. As in previous experiments, minimal photobleaching was observed upon laser excitation, confirming that FRET changes were predominantly target‐induced. In all tested plasma conditions, the addition of 1 µm non‐target RNA (miR342) induced only minor changes in FRET efficiency (6% in 30% plasma, 4% in 50% plasma, and 7% in 100% plasma) (Figure 2c; Figure S1,e,f, Supporting Information). Upon introducing the miR‐21 target, we observed concentration‐dependent changes in FRET efficiency, demonstrating high specificity of the platform. In 30% plasma, FRET change was 25% for 1 µm, 12% for 10 pm, and 8% for 1 pm (Figure S1e, Supporting Information). An estimated LoD of 17.5 pm was found. In 50% plasma, a similar FRET shift was detected 28% for 1 µm, 7% for 10 pm, and 4% for 1 pm (Figure S1f, Supporting Information), with an estimated LoD of 17.81 pm. In 100% plasma, the change in FRET efficiency was 24% for 1 µm, 11% for 10 pm, and 8% for 1 pm with an estimated LoD of 23.42 pm (Figure 2d). These key results are illustrated in Figure 2c, and the remaining histograms are available in (Figure S1, Supporting Information).

### 2.4 Effect of Nucleic Acid Modifications on Hybridization Performance and the Detection Sensitivity of a Biosensor

To address the limitations in target detection posed by the low GC content and reduced thermodynamic stability, or weaker hybridization strength, we systematically investigated the effect of chemical modifications on probe performance within the DNA origami book biosensor system in biofluids. Specifically, we evaluated the impact of incorporating 2′‐F and LNA bases into the lock domain of the biosensor to enhance duplex stability, target affinity, and overall analytical sensitivity. The 2′‐F modifications promote a C3′‐endo sugar pucker, favoring A‐form helix formation and improving duplex stability. Each 2′‐F base has been shown to increase the melting temperature (Tm) by ≈1.8 °C, leading to enhanced hybridization kinetics under physiological conditions.^[^ ^28^ ^]^ Simultaneously, LNA‐modified nucleotides feature a locked ribose conformation, achieved through a methylene bridge between the 2′‐oxygen and 4′‐carbon. This conformational rigidity elevates thermal stability and significantly strengthens hybridization affinity when paired with complementary RNA or DNA strands. Modifications were inserted near the single‐nucleotide polymorphism (SNP) matching region to increase target specificity and enhance thermal stability.^[^ ^29^ ^]^ Two lock variants were engineered: one containing two internal 2‐F modifications (L*) and the other incorporating three LNA bases (L+), with all modifications introduced at the hybridization site. Their performance was evaluated in terms of duplex stability and signal output.

**Figure 3:**
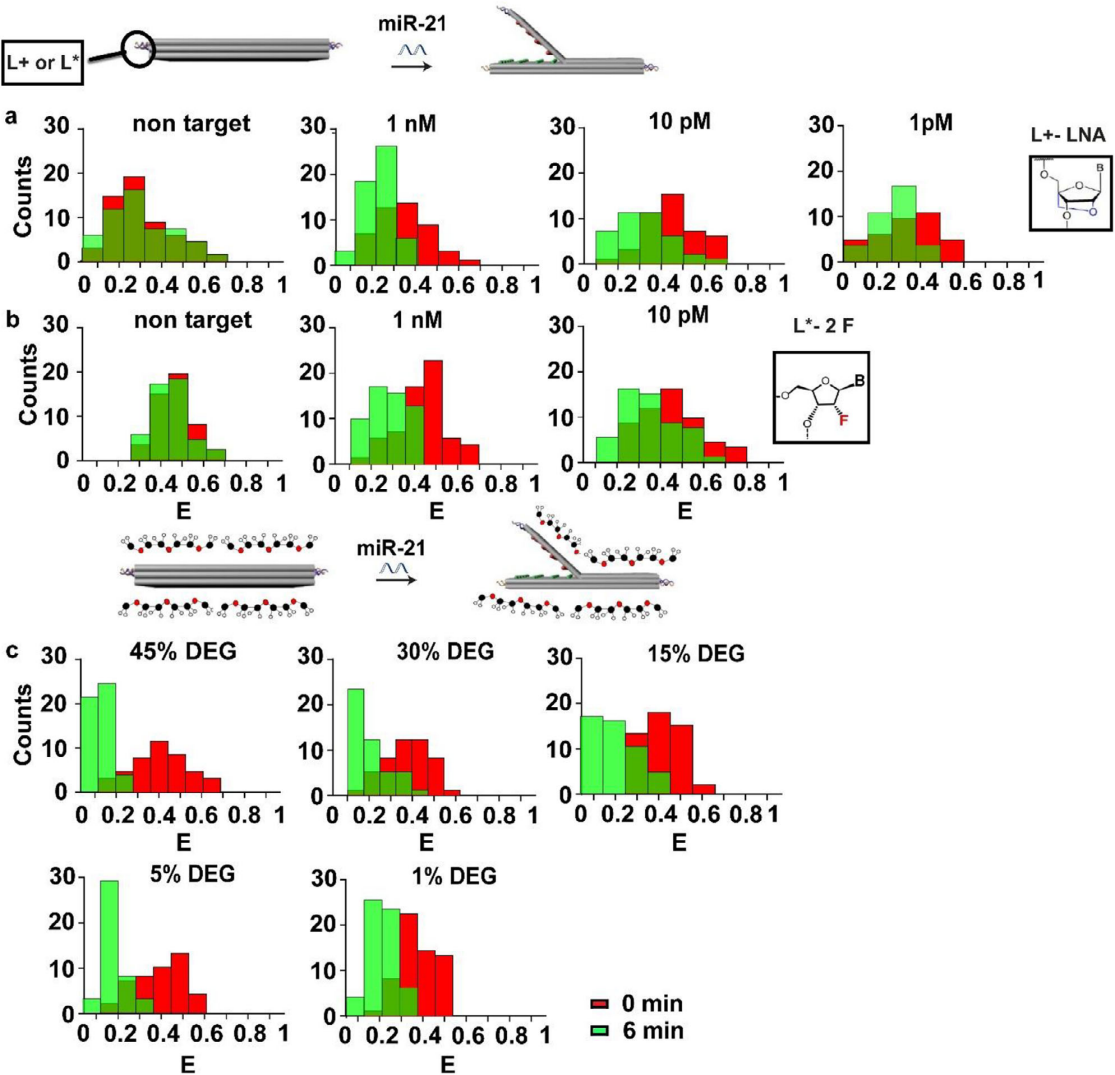
Effect of lock nucleic acid modifications and DEG addition on hybridization efficiency and sensitivity of the DNA origami book biosensor. a) FRET histograms showing detection of spiked miR‐21 using LNA‐modified biosensor L+. b) FRET histograms for detection of miR‐21 using 2F‐modified biosensor L*. c) FRET histograms showing detection of 1 nm miR‐21 with varying v/v% DEG added to the assay. In all panels, a shift from high to low FRET reflects target‐triggered biosensor opening. Note: E represents FRET efficiency, and count is the number of DNA origami biosensors observed.

First, we evaluated target detection in control (buffer) using the DNA book biosensor described above, incorporating modified lock domains into the biosensor structure. The biosensor was tested with miRNA targets at concentrations of 1 µm and 10 pm, employing the DNA origami “book” configuration with lock modifications. A control study with non‐target demonstrated a 1% change in FRET, indicating specificity for L+ and L* locks (Figure 3a,b).

LNA‐modified ‐L+ biosensor demonstrated a FRET efficiency change of 19% for 1 nm and 13% for 10 pm (Figure 3a). In contrast, the 2′‐F–modified L* biosensor exhibited slightly lower FRET shifts of 15% for 1 nm and 10% for 10 pm (Figure 3b). Results indicated that with both modifications and high concentrations of miRNA binding, the performance was similar to that with unmodified locks. However, at lower concentrations, the modified locks bond the target more effectively, as indicated by an ≈3% rise in FRET efficiency. Based on these results, we chose to further examine L+. For the last step of this study, we tested the DNA origami book biosensor with the L+ lock in a control (buffer) at even lower spiked concentrations of 1 pm synthetic miR‐21. The FRET changes at 1 pm showed a 9% decrease. With low spiking, the concentration led to a 2% improvement in signal change when comparing the biosensor with and without L+. These key results are shown in **Figure** **3**a.

### 2.5 Effect of DEG Additive on Performance and Detection Sensitivity of the Book Biosensor

To further enhance the performance of the book biosensor in biofluids, we investigated the impact of polymer additives on miRNA hybridization efficiency. Nordèn et al. previously reported that a matched DNA strand can displace a mismatch strand in a duplex more rapidly in a solution containing non‐ionic semi‐hydrophobic polyethylene glycol (PEG) molecules.^[^ ^30^ ^]^ The authors excluded the possibility that the cause of this effect is PEG‐induced variations in the DNA duplex conformation or duplex melting. Instead, they proposed a hydrophobic effect mechanism comprising three distinct steps. First, interruption of the base stacking and strand invasion; second, stronger hydrogen bonds by decreased water activity; and third, stabilization of the mismatch by intercalation of the hydrophobic crowder into the mismatched gaps.^[^ ^30^ ^]^ In addition, Shi et al. further demonstrated that PEG accelerates non‐enzymatic DNA circuit assembly (hairpin hybridization chain reactions), leading to significantly higher reaction efficiency.^[^ ^31^ ^]^ Specifically, we evaluated DEG, a small polymer structurally related to polyethylene glycol (PEG), as an assay additive. It was shown that DEG influences nucleic acid strand displacement kinetics.^[^ ^32^, ^33^ ^]^ While structurally like PEG, DEG behaves differently: due to its smaller size and semi‐hydrophobic nature, it acts more as a cosolvent than a true molecular crowder. Unlike conventional macromolecular crowding agents, DEG does not produce volume exclusion effects; instead, it promotes hybridization and strand displacement through hydrophobic interactions and direct interactions with nucleobases.^[^ ^32^, ^34^ ^]^

Practically, this could potentially translate to a lower effective LoD in biofluids, consistent with the idea that artificial polymer agents act as hybridization boosters.^[^ ^34^ ^]^ This showcases the potential of the practical usefulness of DEG for both research‐based miRNA detection and clinical applications.

Therefore, DEG was tested at varying concentrations (1%, 5%, 15%, 30%, and 45% v/v) to evaluate its impact on target binding and book biosensor response in the buffer. DNA origami book biosensor structures containing columns (1–3) of Cy3/Cy5 FRET pairs were assembled and immobilized using the same protocol applied in the buffer, serum, and plasma described above. After adding the buffer, we add different concentrations of DEG to the assay. Sensing with 1 µM of target miR‐21, we observed that FRET efficiency decreased by 18% when 1% DEG was present in the assay. The decrease in FRET efficiency was 29% with the use of 5% DEG, 23% with the use of 15% DEG, 23% with the use of 30% DEG, and 30% with the use of 45% DEG. These key results are presented in Figure 3c.

Comparison with the control, where the DNA origami biosensor detected 1 µm of target in the absence of DEG, revealed a 37% decrease in FRET efficiency (Figure S1a, Supporting Information). Results with DEG showed that the DNA origami biosensor maintained robust functionality upon the addition of polymer to the assay. The book biosensor demonstrated effective performance even in highly crowded solutions containing both high concentrations of polymer and target miRNA. Notably, despite potential interference from the crowded environment, a clear and consistent FRET signal change was observed, confirming the operational integrity of the biosensor.

### 2.6 Combined DEG and L+ Lock Further Enhance Biosensor Performance

Encouraged by these results, we hypothesized that the use of DEG in combination with L+ lock might further enhance biosensor performance, particularly at lower target concentrations. To test this hypothesis, we performed experiments using higher DEG concentration (30% and 45%) in combination with L+ lock to detect lower concentrations of miRNA in biofluids.

**Figure 4:**
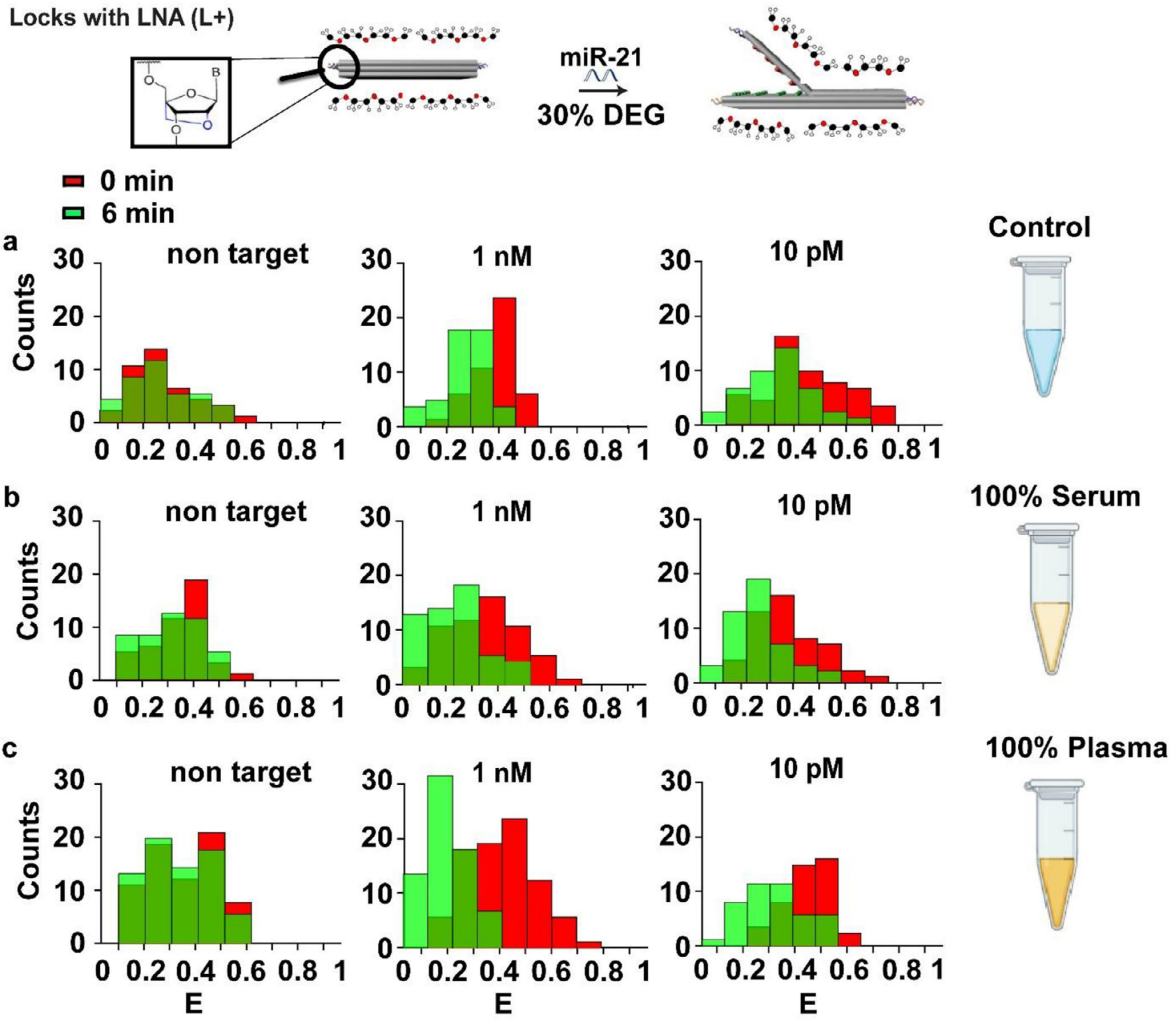
Effect of combined modifications L+ and 30% v/v DEG on hybridization efficiency and sensitivity of the DNA origami biosensor. a) FRET histograms showing detection of spiked miR‐21 using L+ and 30% (v/v) DEG in control (buffer). b) FRET histograms showing detection of spiked miR‐21 using L+ and 30% (v/v) DEG in 100% human serum. c) FRET histograms showing detection of spiked miR‐21 using L+ and 30% (v/v) DEG in human plasma. In all panels, histograms compare the FRET distribution at 0 min (red) and after 6 min of incubation (green). A shift from higher to lower FRET values indicates target‐induced opening of the DNA origami biosensor. Note: E represents FRET efficiency, and count is the number of DNA origami biosensors observed.

Book biosensors with L+ locks were tested in the presence of 30% DEG. Initial tests were conducted in buffer (as a control), followed by a systematic evaluation in undiluted (100%) human serum and human plasma. In each case, the biosensor was spiked with both high (1 µm) and low (10 pm) concentrations of synthetic miR‐21. Control samples with non‐target miR‐342 consistently showed a baseline FRET change of ≈3% in both buffer and biofluids. In a buffer containing 30% DEG, the biosensor L+ exhibited a 16% decrease in FRET at 1 nm miR‐21 and a 12% decrease at 10 pm, confirming the biosensor's sensing capability (**Figure** **4**a). When tested in 100% serum under the same conditions, a 21% decrease of FRET for 1 nM and a 12% decrease in FRET was observed for 10 pM (Figure 4b). Following testing of a book biosensor L+ with 100% human plasma supplemented by 30% DEG, the FRET efficiency decreased by 22% for 1 nm and 16% for 10 pm, indicating enhanced target hybridization (Figure 4c). These results collectively indicate that the integration of LNA‐based probes with DEG as a co‐solvent improves biosensor sensitivity across a range of target concentrations, particularly in undiluted biological fluids such as plasma and serum.

Since we planned to test a high concentration of DEG, we also wanted to determine if using 45% DEG would further enhance sensitivity at low concentrations in biofluids. In the control (buffer), the FRET efficiency changed upon the addition of 1 nm miRNA, resulting in a 22% decrease (Figure S2a, Supporting Information). For a concentration of 10 pm, the FRET decrease was 13% (Figure S2a, Supporting Information). In 100% serum, the FRET reduction for 10 pm was 12% (Figure S2b, Supporting Information). While in 100% plasma, the FRET efficiency decreased by 10% for 10 pm of miR‐21 (Figure S2c, Supporting Information).

Across all tested configurations, the combination of L+ with 30% (v/v) DEG consistently yielded the most favorable results, enhancing signal change at lower target concentrations in both 100% plasma and serum. Although the 45% (v/v) DEG condition also resulted in signal changes, these values were not significantly higher than those observed at 30% (v/v) DEG. Moreover, early results suggest that L+ modification alone modestly improves signal change at low concentrations; however, when paired with DEG, it shows a better signal change in complex matrices.

### 2.7 Performance of the Book Biosensor in Clinical Plasma Samples, for Multiple miRNA Detection using the Fluorescence Quenching Method

Up to this point, our biosensing strategy relied on FRET to monitor conformational changes in the DNA origami biosensor upon target recognition. However, in the subsequent phase of development, we transitioned to a fluorescence quenching‐based detection approach to simultaneously detect two distinct miRNA targets from a single clinical sample. Fluorescence quenching allows multiplexing using spectrally distinct fluorophore‐quencher pairs.

However, before proceeding with multiplexed detection of two miRNAs, we first validated the biosensor's ability to detect a single target, miR‐21, in both spiked and clinical plasma samples. Optimization of the biosensor's sensitivity and performance, including DEG enhancement and origami surface density tuning, was systematically evaluated. Full experimental details and results are presented in the (Figures S3 and S4, Supporting Information).

To evaluate the multiplexing capability of the DNA origami biosensor in a clinically relevant setting, we tested its ability to simultaneously detect two distinct miRNAs directly from plasma samples of breast cancer patients. The biosensor was engineered with (1–2) Cyanine3/ Black Hole quencher 2 (Cy3/BHQ2) dye–quencher columns and L+ on the left side for miR‐21 detection (Cy3 channel), and (1–2) Cyanine5/BlackBerry Quencher 650 (Cy5/BHQ650) dye–quencher columns on the right side for miR‐7a detection (Cy5 channel). This dual‐channel design allowed for the simultaneous and independent detection of both targets within a single assay, demonstrating the biosensor's potential for multiplexing in complex biological fluids.

MiR‐21 and miR‐7a were chosen for their distinct and clinically significant roles in cancer.^[^ ^35^ ^]^ MiR‐21 is a well‐established oncomiR, consistently overexpressed in multiple cancers, including breast and colorectal, and associated with poor prognosis and treatment resistance.^[^ ^36^, ^37^, ^38^ ^]^ In contrast, miR‐7a functions as a tumour suppressor by targeting oncogenic pathways such as EGFR and FAK, thereby inhibiting proliferation and metastasis.^[^ ^39^ ^]^ Their complementary roles make them ideal targets for evaluating the biosensor's diagnostic performance.^[^ ^39^, ^40^ ^]^

As a control experiment, we first validated the system using a mixture of two synthetic miRNA non‐targets (miR‐342 and miR‐153, each at 10 pm), spiked into control (buffer) and healthy plasma. Upon the addition of fluid and non‐target, the biosensor produced a minimal decrease in intensity of 1%–2% in both channels, indicating minimal photobleaching and sequence specificity (Figure S5, Supporting Information).

Then, we performed the same experiment, spiking synthetic miRNA targets (miR‐21 and miR‐7a, each at 10 pM) to induce distinct and simultaneous fluorescence increases in both channels: Cy3 for miR‐21 and Cy5 for miR‐7a. In the buffer, fluorescence signal increases were 17% for the Cy3 channel (miR‐21) and 12% for the Cy5 channel (miR‐7a) (Figure S5, Supporting Information). For plasma, we observed a 12% increase in the Cy3 channel (miR‐21) and a 10% increase in the Cy5 channel (miR‐7a) (Figure S5, Supporting Information). These results confirm the biosensor's stability and multiplexing capability across diverse biological media.

Next, we evaluated the multiplexing performance of the biosensor by simultaneously detecting miR‐21 and miR‐7a within total small RNA extracts from plasma. Small RNA was isolated from plasma Patient 5 (0.0107 ng µL^−1^) and 6 (− 0.0243 ng µL^−1^) and subsequently diluted to a final working volume of 300 µL. These samples were then used to assess biosensor performance in a controlled, RNA‐specific context. For more accurate statistical analysis, we combine the data into a single histogram (Figure S6c, Supporting Information). From this combined histogram, the results showed that the intensities of Cy3 and Cy5 increased by 8% and 9%, respectively. For clarity and transparency, individual histograms for each patient sample are presented separately in the Figure S6a,b (Supporting Information).

**Figure 5:**
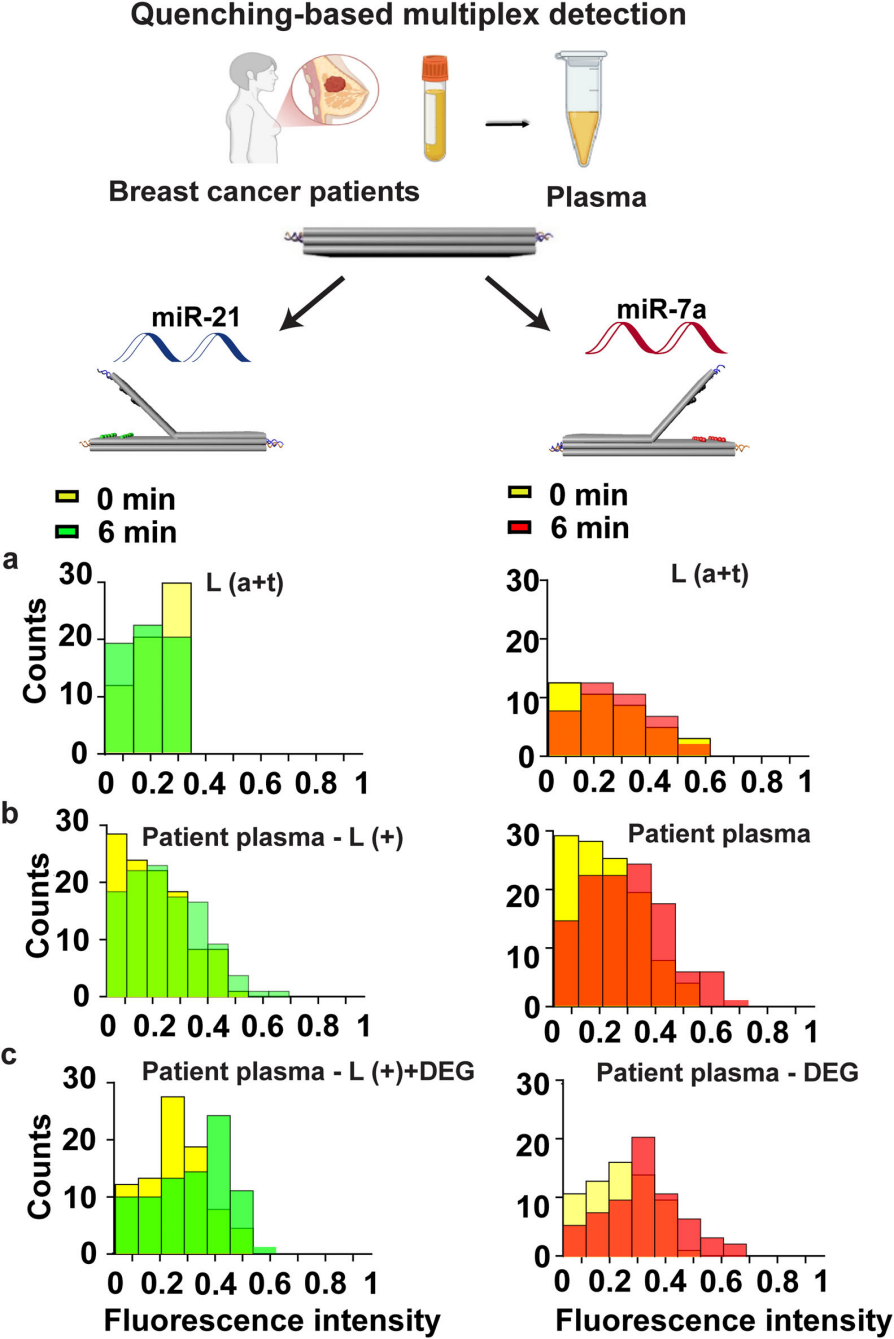
Multiplex detection of miR‐21 and miR‐7a in clinical plasma samples using the quenching‐based DNA origami biosensor. The left panel shows the detection of miR‐21 in plasma samples, while the right panel shows the detection of miR‐7a. Each set of histograms is arranged in three rows: a) the top row represents detection in patient plasma with the modified control locks L(a+t), b) the middle row with the modified lock L+ on the left side of DNA origami biosensor in patient samples, and c) the bottom row with the modified lock L+ on the left side of DNA origami biosensor in patient samples and the presence of 30% (v/v) DEG. In all cases, the red and green histograms indicate the increase in fluorescence intensity following the addition of the target miRNA, reflecting the opening of the DNA origami structure. The yellow histograms correspond to the fluorescence intensity in the closed state of the biosensor.

The final multiplexing study aimed to evaluate the system with patient plasma samples. To demonstrate the high specificity of our DNA origami system, we designed a set of non‐complementary control lock strands, consisting of homopolymeric sequences (poly‐T and poly‐A, e.g., TTT and AAA), which we refer to as L(A+T). The assay was repeated using plasma from Patients 8 and 9. Under these conditions, a decrease of signal by 2 to 3% was observed in both detection channels, indicating minimal photobleaching and sequence specificity in complex biological samples (**Figure** **5**a).

Once we confirmed the specificity of our book biosensor, we attempted to detect miR‐21 and miR‐7a in 4 different plasma samples from patients using our book biosensor. A histogram combining data from all four plasma samples revealed that Cy3 fluorescence increased by 12% (miR‐21) and Cy5 fluorescence increased by 11% (miR‐7a) (Figure 5b). Individual patient samples exhibited consistent signal changes, demonstrating the biosensor's reproducibility across clinical samples. For clarity and transparency, detailed fluorescence responses and individual histograms are provided in the (Figure S7a,c,d,f, Supporting Information).

Next, we aimed to enhance the sensitivity of our book biosensor by adding 30% DEG to the assay and testing with plasma from Patients 7 and 9. In the histogram (Figure 5c), combining all collected data, the intensities of Cy3 and Cy5 increased by 17% and 14%, respectively. Individual patient samples exhibited consistent signal changes, demonstrating the biosensor's reproducibility across clinical samples. For clarity and transparency, detailed fluorescence responses and individual histograms are provided in the (Figure S7b,e, Supporting Information).

## 3 Conclusion

In this study, we demonstrate that our DNA origami book biosensor enables efficient detection of low‐GC‐content (35%) miR‐21 directly in undiluted (100%) biofluids. The calculated limits of detection (LoD) reached 0.69 pm in buffer with LNA modification, 8.86 pm in 100% serum, and 23.24 pm in 100% plasma. A summary of all calculated LoDs is found in **Table** **1**.

**Table 1:** Limit of Detection (LoD) for different sample matrices.

Sample Matrix	LoD [pm]
Control / buffer	4.55
30% Serum	6.87
50% Serum	7.94
100% Serum	8.86
30% Plasma	17.15
50% Plasma	17.81
100% Plasma	23.42
LNA + Control	0.69

Furthermore, we observed a notable increase in fluorescence intensity during the detection of miR‐21 and miR‐7a in plasma samples, confirming the sensor's functionality in complex biological environments and its multiplexing capability. However, larger numbers of miRNA should be experimentally validated. The further increase in fluorescence was additionally improved by the addition of DEG as an additive in our assay and LNA lock modification. These results suggest that when a DNA strand contains LNA modifications, its base‐pairing fidelity and strand displacement kinetics increase. These findings are consistent with previously reported properties of the LNA modification introduced into a DNA sequence to enhance target binding specificity.^[^ ^41^, ^42^, ^43^ ^]^ Additionally, our findings demonstrate the practical value of DEG as a hybridization‐enhancing cosolvent. By accelerating strand displacement and stabilizing duplex formation, DEG improves detection sensitivity in complex biofluids. These artificial polymer potentials act as hybridization boosters.^[^ ^34^ ^]^ This showcases the practical usefulness of DEG for both research‐based miRNA detection and clinical applications. Moreover, we demonstrated that our biosensor is capable of detecting microRNAs under clinically relevant conditions; however, the targets identified here are typically among the most abundant miRNAs in human plasma. As such, we remain cautious regarding the platform's sensitivity to low‐abundance targets, a critical consideration for early disease detection. Nevertheless, the biosensor provides several notable advantages. It employs a streamlined, single‐step workflow in which the biosensor is mixed with the sample and fluorescence is measured, eliminating the labor‐intensive RNA extraction or amplification steps typically required for qPCR or sequencing.

To contextualize these findings relative to established diagnostic standards, we compared our biosensor with qRT‐PCR and next‐generation sequencing (NGS). While qRT‐PCR achieves femtomolar to attomolar sensitivity, it relies on enzymatic amplification, multistep protocols, and specialized laboratory infrastructure. NGS, although comprehensive for small RNA profiling, is costly, time‐intensive, and unsuitable for rapid diagnostics. In contrast, our biosensor directly detects low‐GC‐content miRNAs, such as miR‐21, in undiluted biofluids without the need for amplification. The assay delivers results in under 10 min, supports multiplexed detection, and operates as an actual one‐step protocol. It can be customized to simultaneously screen panels of disease‐specific miRNAs from small blood volumes, offering improved diagnostic accuracy over single‐marker.^[^ ^38^ ^]^ Biosensors can also differentiate single‐base mutations or family miRNA variants by design of the hybridization locks, addressing a key challenge in miRNA diagnostics where sequence homology is high. The self‐assembly of the DNA origami biosensor further enables cost‐effective and scalable production. When combined with microfluidic systems, the platform could achieve sensitivities in the femtomolar range, collectively positioning it as a strong candidate for next‐generation miRNA diagnostics.

Beyond clinical translation, the platform programmability, low picomolar‐level sensitivity, and a portable optical readout also show promise as a versatile research tool, offering an accessible complement or alternative to qRT‐PCR and NGS, while enabling deployment in point‐of‐care settings. Future work will benchmark this technology against qRT‐PCR and NGS in larger clinical cohorts to validate its translational potential. Taken together, these advances establish DNA origami biosensors as a promising foundation for scalable, precise, and accessible diagnostics, while also offering a powerful research platform for elucidating miRNA function and regulation.

## 4 Experimental Section

### Design and Assembly of a DNA Origami Book Biosensor

The structure was designed using caDNAno software (https://cadnano.org/).^[^ ^44^ ^]^ The file generated by caDNAno software was submitted to CanDo (Computer‐aided engineering for DNA origami, https://cando‐dna‐origami.org/) for further evaluation of the shape, flexibility, and dynamics. The assembly of the DNA origami book biosensor is achieved by mixing 10 nm single‐stranded scaffold DNA (type: p8064, Europhins MWG Operon, Ebersberg, Germany) with unmodified staple strands (100 nm each) (HPSF purified, LubioScience Switzerland IDT, Zurich, Switzerland) and 1 µM of modified staple strands with fluorophores (ddUTP‐Cy3 and ddUTP‐Cy5, Jena Bioscience, Germany), oligonucleotides with bh2 and bbq650q (Biomers, Ulm, Germany), and 1 µm of biotin‐modified oligonucleotides (Biomers, Ulm, Germany). All modified oligonucleotides were purified by HPLC. The sequences of all oligonucleotides used in this study are reported in Domljanovic et al.^[^ ^19^ ^]^ Additional modifications to locks (LNA and 2F) studied and implemented in this study are listed in the in Table S2 (Supporting Information). The annealing of the scaffold DNA and staple strands mixture was performed using the temperature ramp method: heating the solution to 80 °C for 5 min, cooling to 65 °C over the first 15 min, and then cooling further to 4 °C over 16 h.

### Purification of Assembled DNA Origami

DNA origami book biosensor structures were assembled with staple strands, and purification of unincorporated staple strands was done with two different methods. The first method was based on 100 kDa Amicon Ultra‐0.5 mL centrifugal filters (Millipore, Germany). Amicon filters were pre‐wet by filling with an annealing buffer (1× TAE/10 mm Mg^2+^) with 500 µL and centrifuged at 8000 g for 10 min. Then, the samples were loaded onto the filter (100 µL) and completed to 500 µL with the annealing buffer. The sample was centrifuged at 8000 g for 8 min and washed 4 times with 400 µL of annealing buffer. The second method used for purification was agarose gel electrophoresis. The assembled book biosensor structure (100 µL) sample and 6× glycerol (20 µL) were loaded into a 1% agarose gel. The gel was run at 70 V for 1 h, and the corresponding band of fluorescently labeled structures was cut out from the gel using a razor blade and extracted by squeezing with a glass slide.

### Single‐Origami Detection by Wide‐Field Fluorescence Microscopy

The 12‐well glass slide (Ibidi, Germany) with removable silicon chambers was incubated with 50 µL of 0.5 mg mL^−1^ biotinylated bovine serum albumin solution (Sigma‐Aldrich, Buchs, Switzerland) for 15 min at room temperature, washed three times with 1× TAE/12 mm Mg^2+^, and incubated with 50 µL of 0.5 mg mL^−1^ neutravidin solution (Thermo Fisher Scientific, Basel, Switzerland) for 15 min and washed with 1× TAE/12 mm Mg^2+^. Biotinylated DNA origami book samples (100 pM in 1× TAE/12 mm Mg^2+^) were incubated for 15 min and washed three times with 1× TAE/12 mM Mg^2+^. The mix of the oxygen scavenging system, consisting of a 1:1:1 ratio of 1× PCA, 1× PCD, and 1× Trolox mix in 1× TAE/12 mM Mg^2+^ was added into the glass slide chambers where structures were functionalized. The oxygen‐scavenging system was prepared from stock solutions before use. The 100× Trolox stock solution contains 100 mg of Trolox, 430 µL of methanol, and 345 µL of NaOH (1 m) in 3.2 mL of water. The 40× PCA stock solution contains 154 mg of PCA in 10 mL of water (pH 9.0). The 100× PCD stock solution contains 9.3 mg of PCD and 13.3 mL of buffer (50% glycerol stock in 50 mm KCl, 1 mM EDTA, and 100 mm Tris HCl, pH 8.0). Experiments were performed on a home‐built TIRF widefield microscope, based on an inverted Olympus IX83 body. Laser lines of 532 and 640 nm were used (gem, Laser quantum), filtered by a clean‐up filter (ZET532/640×). Dichroic Mirrors (DM) were used to join the path, and Pol + λ/4 is included for circular polarization. The laser light was then focused by two lenses (AC508‐100‐A‐ML and AC254‐030‐A‐ML, Thorlabs, Germany) into the back focal plane of the UPLAPO100xOHR (1.5 NA, Olympus) objective. After, a Laser Dual Band Set (ET‐532/640 nm, DM3) was used to excite the sample and filter the emission simultaneously. The emission was split into two channels using DM4 (T635lpxr) into an Optosplit II bypass (Cairn) system, to finally reach the chip of the CMOS camera (C14440 ORCA‐Fusion, Hamamatsu). To image the structures using FRET‐based detection, a 532 nm laser at 1.0 mW was employed for Cy3. For structures with quenching‐based detection, lasers at 532 and 640 nm at 1.0 mW were used for Cy3 and Cy5, respectively, with direct excitation. After excitation, images were taken every 2 min for 10 min, and data points were collected using two channels: the first for Cy3 emission and the second for Cy5 emission. For the quenching‐based detection mechanism, the direct excitation of Cy3 or Cy5 was collected using the respective channels. Data analysis was performed on a custom‐developed Python software. With this software, an individual structure was identified, fit it using a 2D Gaussian model at the center of the section, and extract its intensity from all relevant pixels in each image. The energy‐transfer efficiency for single donor‐acceptor pairs was calculated by:

(1) $$E=\frac{I_{A}}{I_{A}+I_{D}}$$

where *E* is the energy‐transfer efficiency, *I_A_* is the acceptor intensity, and *I_D_* is the donor intensity.^[^ ^17^ ^]^ For quenching‐based detection, the increase in fluorescence intensity was calculated by subtracting the intensity at the 0 min period from the intensity at 6 min. The difference between the two intensities was then divided by the intensity at 0 min of the same DNA origami book biosensor.

### Isolation of miRNA from Human Plasma

To identify circulating miRNAs in human blood samples, 400 µL of four distinct plasma samples from breast cancer patients was utilized. miRNAs were extracted using the miRNeasy Serum/Plasma Advanced Kit (cat. no. 217204‐QIAGEN). Previously collected Plasma samples from patients with primary, non‐metastatic breast cancer were used in this study, Cattin et al.^[^ ^45^ ^]^ Ethical approval for sample collection and use was obtained from the Cantonal Ethics Commission for Human Research on Humans in Canton Ticino (CE 2967) and the CERVD Ethics Commission in Lausanne extended to Vaud, Fribourg, and Neuchâtel.

### Healthy Human Plasma and Serum

Normal human serum (Sigma–Aldrich, H6914) and human plasma (Sigma–Aldrich, P95230) were used. These biofluids were utilized in spiking experiments in which different synthetic miRNAs of interest were added and detected.

### Synthetic Polymer

To study the influence of polymers on the detection of miRNA, diethylene glycol dimethyl ether (DEG), which was also purchased from Sigma–Aldrich was used.

## Conflict of Interest

The authors declare no conflict of interest.

## Author Contributions

I.D. and C.R. conceptualized the project and designed the experiments. I.D. performed the microscopy experiments, acquired, and analyzed the data. S.K. designed the DNA origami book biosensor. All of the authors interpreted and discussed the acquired data. I.D. wrote and prepared the original draft of the manuscript. C.R., S.K., and G.P.A. reviewed and edited the manuscript. All authors have read and agreed to the published version of the manuscript.

## Supporting information



Supporting Information

## Data Availability

The data that support the findings of this study are available from the corresponding author upon reasonable request.
