# Supplementary material for: Enhancing the miRNA Detection Sensitivity of DNA Origami Book Biosensors Using Lock Modifications and a Polymer Additive
Source: Small. 2025 Nov 14;21(52):e12100. doi: 10.1002/smll.202512100 (PMC12747634; doi:10.1002/smll.202512100)
Supplement: Supplementary file 1 — Supporting Information [file SMLL-21-e12100-s001.docx]

Supplementary Information

Enhancing the miRNA detection sensitivity of DNA origami book biosensors using lock modifications and a polymer additive

Ivana Domljanovic^1,3 *^, Samet Kocabey^1,2^, Guillermo P. Acuna^2,3^, and Curzio Ruegg^1,2,4, *^

1. Laboratory of Experimental and Translational Oncology, Department of Oncology, Microbiology and Immunology, Faculty of Science and Medicine, University of Fribourg, Chemin du Musée 18, PER17, 1700 Fribourg, Switzerland.

2. NCCR Bio-inspired Materials, University of Fribourg, 1700, Fribourg, Switzerland

3. Photonic Nanosystems, Department of Physics, Faculty of Science and Medicine,

University of Fribourg, Chemin du Musée 3, PER08, 1700 Fribourg, Switzerland.

4. XEMPERIA SA, Marly Innovation Center, Rue de l’Ancienne Papeterie 180, 1723 Marly, Switzerland.

* Correspondence to: [curzio.ruegg@unifr.ch](mailto:curzio.ruegg@unifr.ch) and [ivana.domljanovic@unifr.ch](mailto:ivana.domljanovic@unifr.ch)

**Table of Contents**

**Figure S1.** FRET-based detection of miR-21 using DNA origami book biosensors across different biofluids.................................................................................................................................................. 3

**FigureS2.** Effect of 45% (v/v) DEG on the sensitivity of the DNA origami book biosensor…………………… 6

**Figure S3.** Quenching-based detection of synthetic miR-21 and miR-21 from extracted small RNA using a DNA origami biosensor........................................................................................................................ 7

**Figure S4.** Quenching-based detection of miR-21 from breast cancer patient plasma samples using a DNA origami biosensor……………………………………………………………………………………………………………………. 8

**Figure S5.** Multiplex detection of synthetic miR-21 (Cy3, green) and miR-7a (Cy5, red) in buffer and 100% human plasma using a quenching-based DNA origami biosensor…………………………………………… 10

**Figure S6.** Multiplex detection of miR-21 (Cy3, green) and miR-7a (Cy5, red) from small RNA extracted from breast cancer patient plasma using a quenching-based DNA origami biosensor.......................... 11

**Figure S7.** Multiplex detection of miR-21 (Cy3, green) and miR-7a (Cy5, red) directly from breast cancer patient plasma using a quenching-based DNA origami biosensor, with and without DEG.……………….. 13

**Table S1.** List of the target oligonucleotide sequences....................................................................... 15

**Table S2.** Modified lock sequences for miR-21 detection (left side of the DNA origami biosensor...... 15

**Table S3.** Mapping of patient IDs to tube labels and sample information........................................... 16

**Figure S1: FRET-based detection of miR-21 using DNA origami book biosensors across different biofluids.** (a) Representative histograms of FRET efficiency distributions for DNA origami book biosensors incubated in control buffer after exposure to synthetic miR-21 at 1 µM and 100 pM concentrations. Histograms illustrate the shift from the closed (high-FRET, red, 0 min) to open (low-FRET, green, 6 min) state, indicating a conformational change upon target recognition. (b) The same experimental conditions were applied in 30% human serum. Full dataset shown. (c) The same experimental conditions were applied in 50% human serum. Full dataset shown. (d) The same experimental conditions were applied in 100% human serum. Only results for 1 µM and 100 pM miR-21 are shown, which were not included in the main manuscript. (e) The same experimental conditions were applied in 30% human plasma. Full dataset shown. (f) The same experimental conditions were applied in 50% human plasma. Full dataset shown. (g) The same experimental conditions were applied in 100% human plasma. Only results for 1 µM and 100 pM miR-21 are shown, which were not included in the main manuscript. (h–i) Summary plots showing dose-dependent ΔFRET responses in 30% and 50% human serum and plasma. Data were analysed using log-linear regression across the dynamic range of concentrations, followed by linear regression in the lower concentration range. Note: E represents FRET efficiency; count indicates the number of DNA origami biosensors analysed.


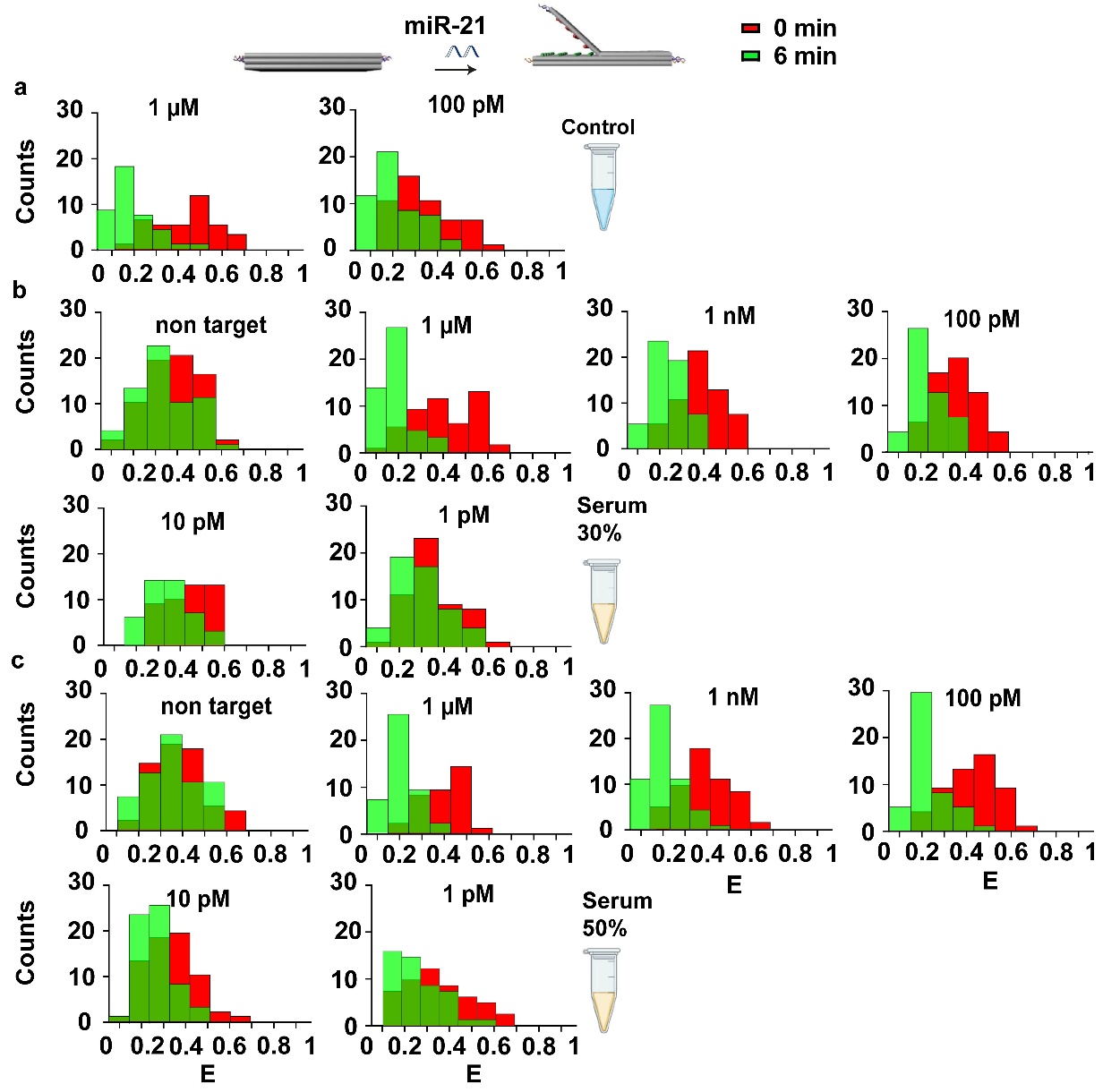


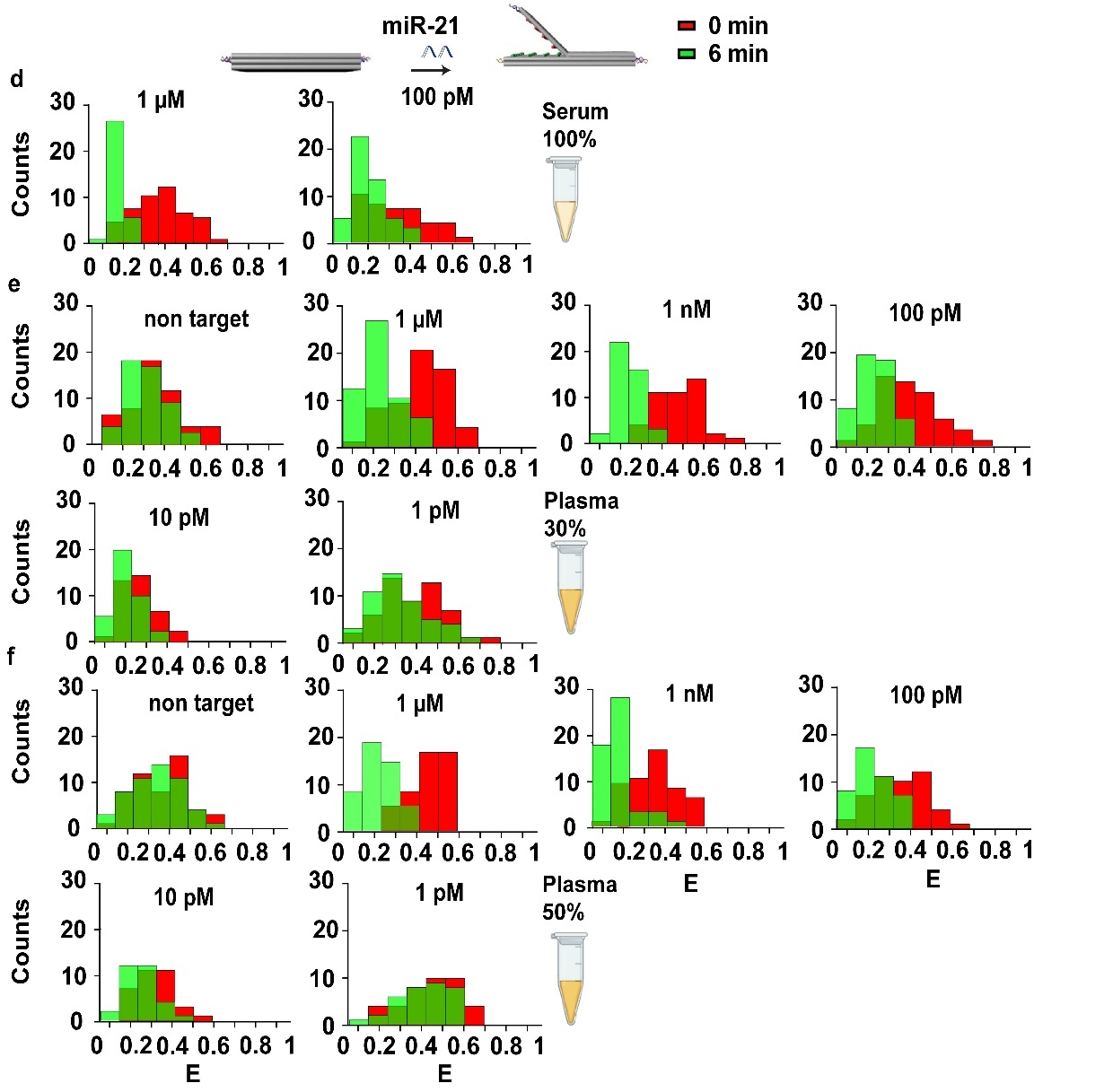


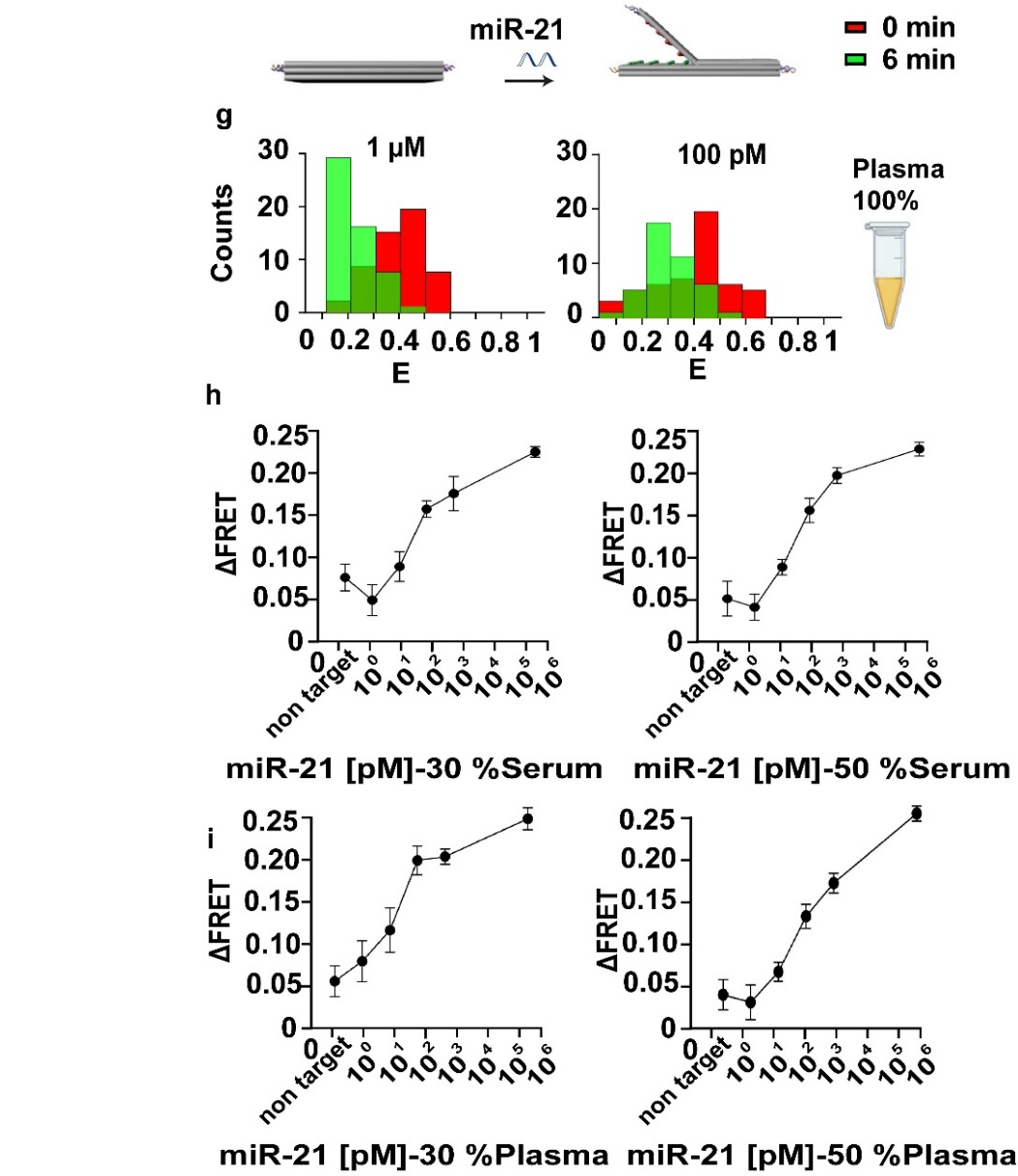


**Figure S2. Effect of 45% (v/v) DEG on the sensitivity of the DNA origami book biosensor.** (a) FRET efficiency histograms showing detection of synthetic miR-21 at 1 nM and 10 pM concentrations in control buffer, with 45% (v/v) DEG added to the assay. (b) FRET efficiency histograms showing detection of 10 pM synthetic miR-21 in 100% human serum with 45% (v/v) DEG added. (c) FRET efficiency histograms showing detection of 10 pM synthetic miR-21 in 100% human plasma with 45% (v/v) DEG added. In all panels, a shift from high FRET to low FRET indicates biosensor opening upon target recognition. Note: *E* refers to FRET efficiency; *count* refers to the number of DNA origami biosensors analyzed.


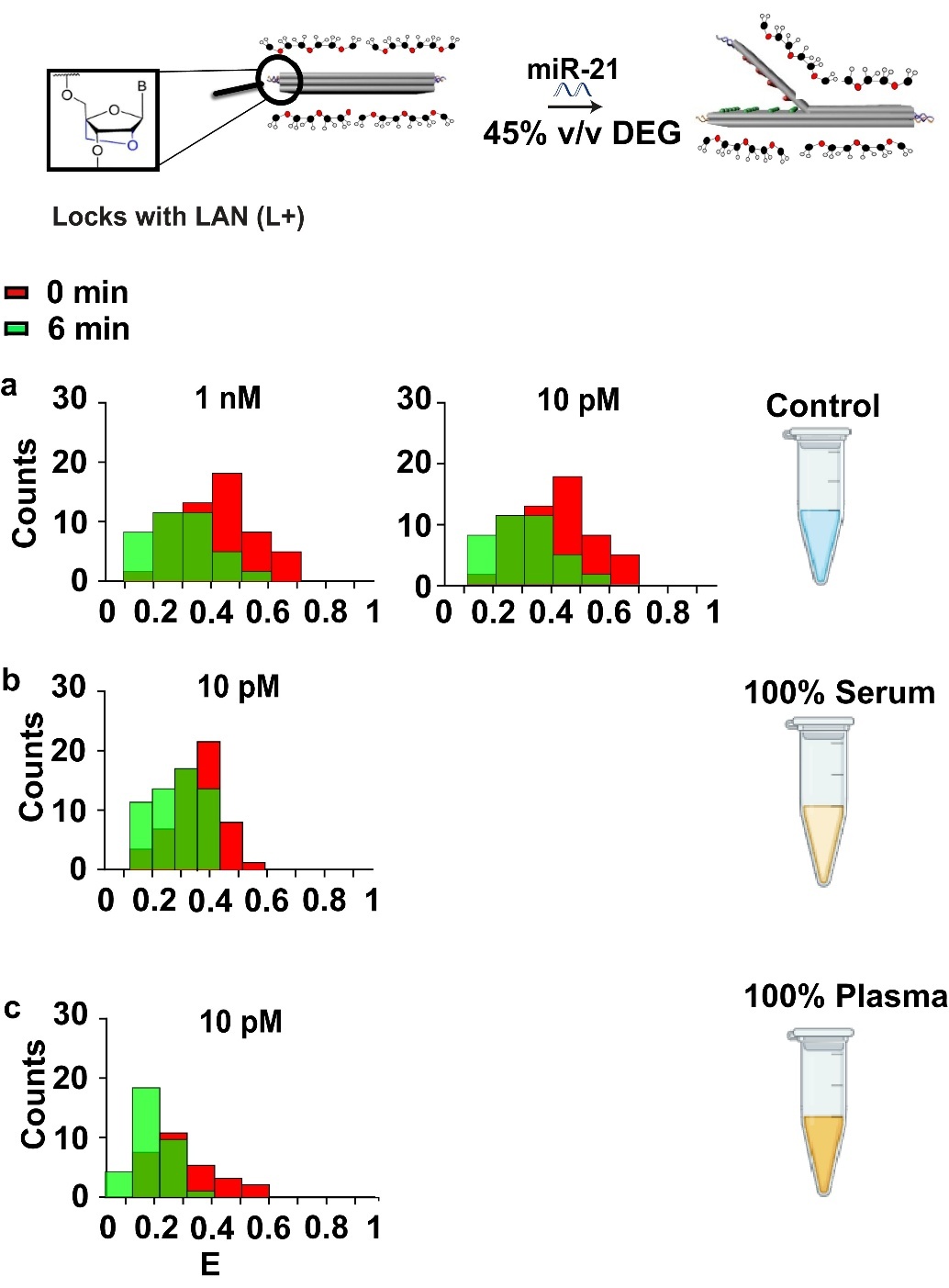


**Figure S3. Quenching-based detection of synthetic miR-21 and miR-21 from extracted small RNA using a DNA origami biosensor.** The biosensor used is a DNA origami book with two columns (1–2) of Cyanine 3/Black Hole Quencher 2 (Cy3/bhq2) dye-quencher pairs on the left side and L+ targeting miR-21, following the protocol from earlier experiments. As our first step, we conducted an experiment in which we spiked 10 pM of miR-21 in plasma to demonstrate the principle. We observed a 12% increase in fluorescence (Figure S3a). Followed by the evolution of the biosensor’s ability to detect miR-21 from total small RNA extracted from plasma samples. Small RNA was isolated from patient plasma samples, Patient 1 and 2, and subsequently diluted to a final working volume of 300 µL. This resulted in an approximate RNA concentration of 0.27 ng/µL for Patient 1 and 1.96 ng/µL for Patient 2. Results showed an increase in fluorescence for Patient 1 (12%) and Patient 2 (14%). All fluorescence intensities have been compiled into a single histogram for comparison (Figure S3c). At the same time, individual sample data are shown in Figure S3b. In all panels, yellow histograms represent the closed (quenched) state of the biosensor at 0 min, while green histograms represent the open (fluorescent) state at 6 min, reflecting successful miR-21 recognition.


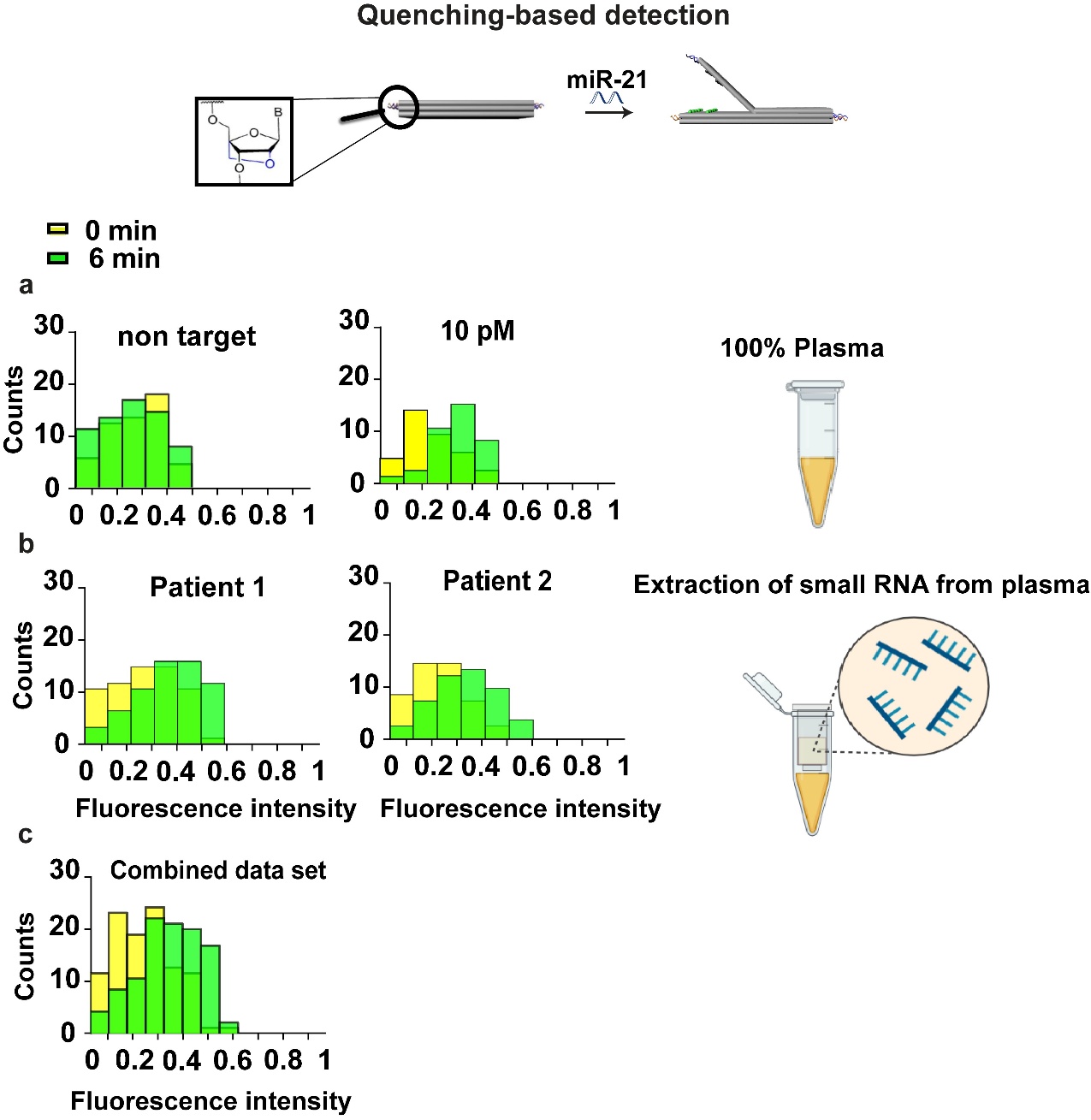


**Figure S4. Quenching-based detection of miR-21 from breast cancer patient plasma samples using a DNA origami biosensor.** Detection of miR-21 from clinical samples followed. Upon the addition of 200 µL of breast cancer plasma from Patient 3, a 10% increase in fluorescence intensity was observed within 6 minutes. We then repeated the same experiment using plasma from a different breast cancer patient, Patient 4, which resulted in a 12% increase in fluorescence signal. All fluorescence changes have been compiled into a single histogram for a better overview of all data (Figure S4c), and individual sample data are provided in the Supplementary Information, Figure S4a and b.

To further enhance sensitivity, we repeated the experiment using the same plasma samples supplemented with 30% DEG. This modification resulted in an 18% increase in fluorescence intensity for Patient 3 and a 16% increase for Patient 4. To conclude the addition, we observed an increase in signals for 8% (Patient 3) and 6% (Patient 4 ) after adding the polymer (Figure S4a and b). These results demonstrate that DEG can enhance biosensor performance in clinically relevant samples by improving target accessibility and signal strength.

To further improve detection sensitivity, we investigated whether the surface density of DNA origami structures influenced the biosensor’s ability to detect low concentrations of miR-21 in patient plasma. We hypothesized that reducing the surface density could minimize steric hindrance and enhance hybridization efficiency in complex biological matrices. Therefore, we decreased the DNA origami incubation concentration from approximately 100 pM (high-density condition) to 25 pM (low-density condition), thereby reducing the number of immobilized structures per field of view. This adjustment allowed us to test whether sparser surface loading improves target accessibility and signal transduction under physiologically relevant conditions.

Under these conditions, we observed a 16% increase upon adding the same plasma samples as above, indicating that the increase in fluorescence was approximately 6.5% (Figure S4b - Patient 4 !) Then, we repeated this experiment and added 30% v/v DEG to a patient sample; the fluorescence signal increased by 25%, representing an intensity increase of 9% once DEG was added to the assay (Figure S4b - Patient 4 ! + DEG). Suggesting that the combination of low origami density and DEG co-solvent conditions can synergistically improve signal response and overall biosensor sensitivity in plasma.

**
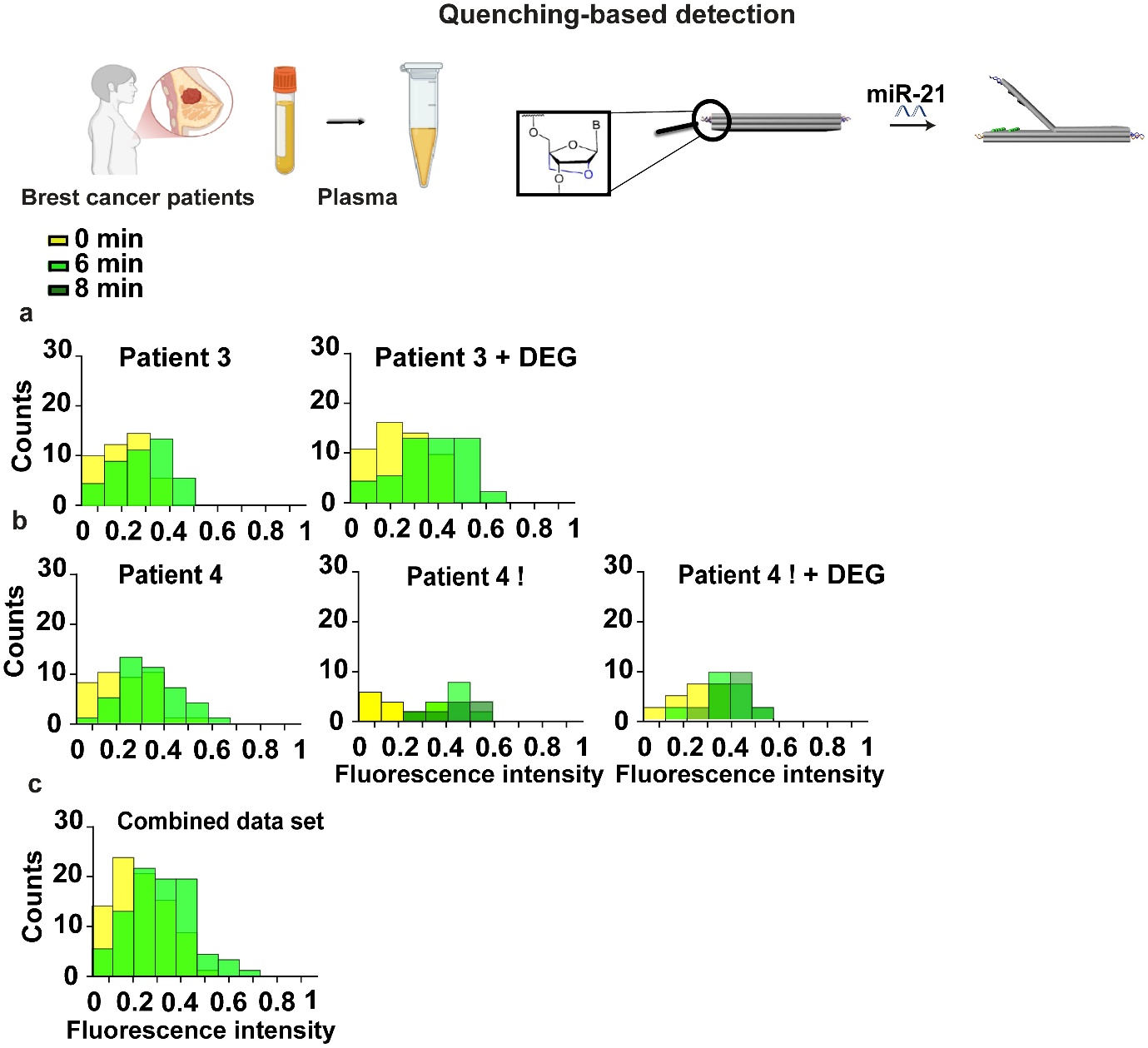
**

**Figure S5. Multiplex detection of synthetic miR-21 (Cy3, green) and miR-7a (Cy5, red) in buffer and 100% human plasma using a quenching-based DNA origami biosensor.** DNA origami biosensors were designed for the simultaneous detection of two miRNAs. The left side of the structure incorporated 1–2 columns of Cy3/BHQ2 pairs with L+ modification for miR-21 detection, while the right side included 1–2 columns of Cy5/bb650Q pairs for miR-7a detection. Target binding induced structural opening of the biosensor, resulting in increased fluorescence intensity. (a) Histograms showing fluorescence intensity changes upon detection of synthetic miR-21 and miR-7a (10 pM each) spiked into the control (buffer), along with non-target controls. (b) Histograms showing detection of synthetic miR-21 and miR-7a (10 pM each) spiked into 100% human plasma, along with non-target controls. In all panels, yellow histograms represent the closed (quenched) state of the biosensor at 0 min. In comparison, green (Cy3) and red (Cy5) histograms at 6 min represent increased fluorescence following target binding and biosensor activation. Note: Fluorescence intensity corresponds to biosensor opening; counts refer to the number of individual DNA origami biosensors analyzed.


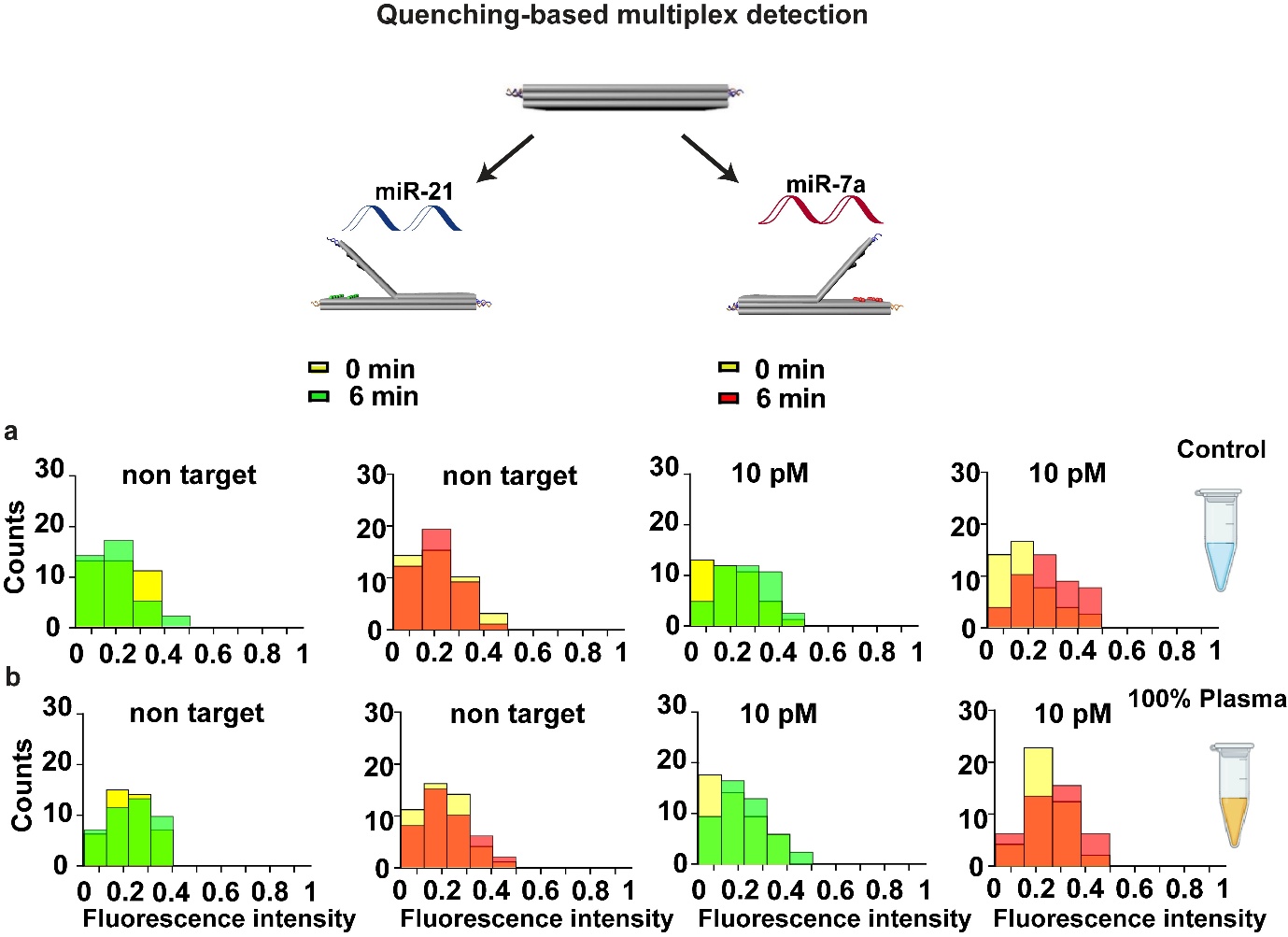


**Figure S6. Multiplex detection of miR-21 (Cy3, green) and miR-7a (Cy5, red) from small RNA extracted from breast cancer patient plasma using a quenching-based DNA origami biosensor.**
DNA origami biosensors were engineered for the simultaneous detection of two miRNAs. The left side of the structure contained 1–2 columns of Cy3/BHQ2 donor–quencher pairs with L+ modifications for miR-21 detection, while the right side included 1–2 columns of Cy5/bb650Q pairs for miR-7a detection. Target binding induced biosensor opening, resulting in increased fluorescence. (a) Histograms showing fluorescence intensity changes following detection of miR-21 and miR-7a from small RNA extracted from breast cancer plasma of Patient 5. (b) Histograms showing detection from Patient 6, using the same biosensor configuration. (c) Combined histograms presenting merged data from both patient samples, reflecting the overall detection performance of the biosensor. In all panels, yellow histograms represent the closed (quenched) state at 0 min, while green (Cy3 for miR-21) and red (Cy5 for miR-7a) histograms at 6 min represent fluorescence increases due to target-induced biosensor activation. Note: Fluorescence intensity corresponds to biosensor opening, and counts indicate the number of individual DNA origami biosensors analyzed.


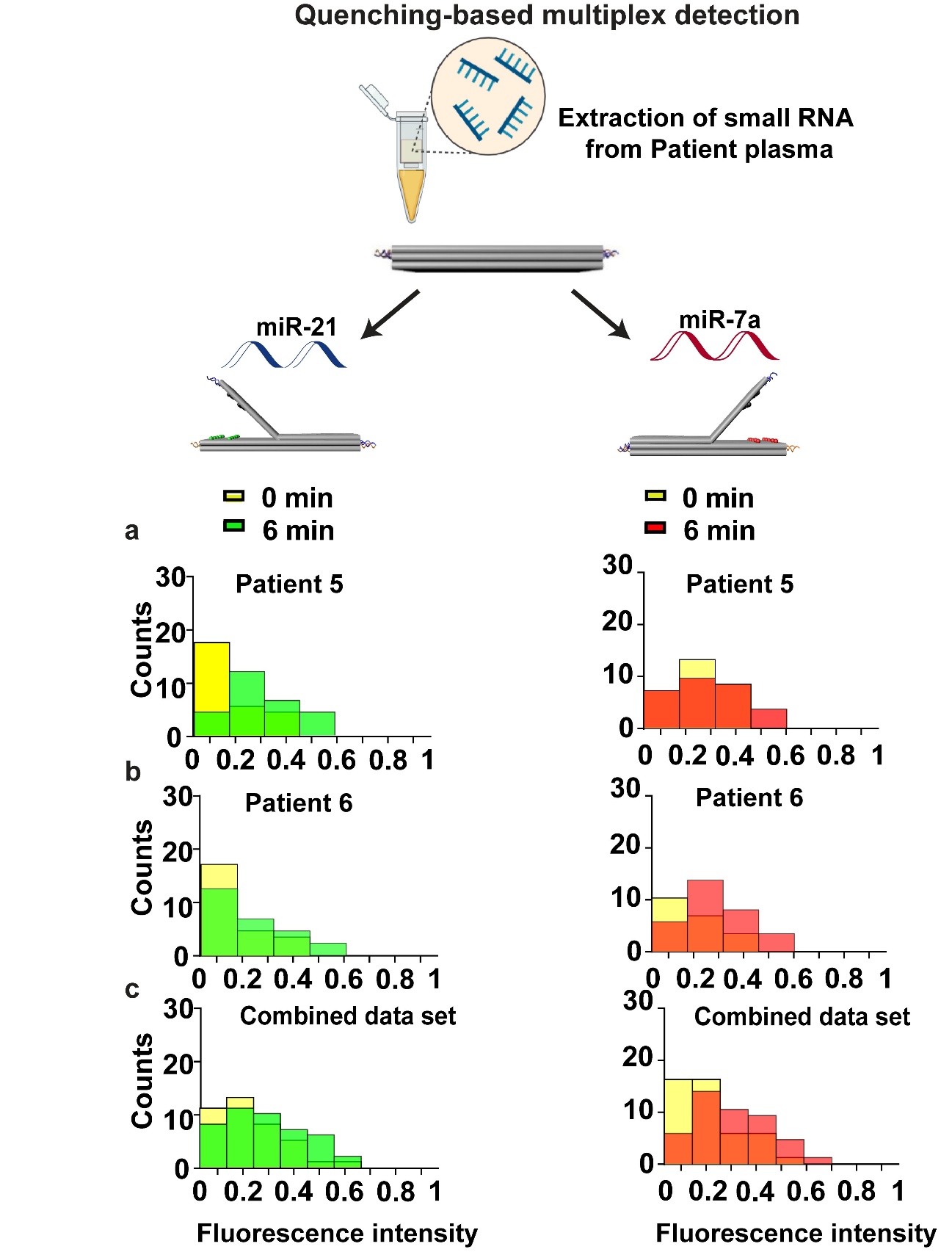


**Figure S7. Multiplex detection of miR-21 (Cy3, green) and miR-7a (Cy5, red) directly from breast cancer patient plasma using a quenching-based DNA origami biosensor, with and without DEG.** DNA origami biosensors were designed for the simultaneous detection of miR-21 and miR-7a. The left arm of the biosensor incorporated 1–2 Cy3/BHQ2 donor–quencher pairs with L+ modifications for miR-21 detection, and the right arm included 1–2 Cy5/bb650Q pairs for miR-7a detection. Binding of target miRNAs triggers structural opening of the biosensor, leading to increased fluorescence. (a) Detection of miR-21 and miR-7a directly from the plasma of Patient 7 (1211.67ng/µL of total RNA) exhibited a 14 % increase in Cy3 and a 11% increase in Cy5. (b) Patient 7 with 30% v/v DEG added to enhance assay sensitivity. Results showed the increases were 16% (Cy3) and 11% (Cy5). (c) Detection from Patient 8 (1248.13 ng/µL of total RNA) displayed a 13% increase in Cy3 and a 10 % increase in Cy5). (d) Detection of plasma of Patient 9 (1248.33ng/µL of total RNA) showed increases of 11 % (Cy3) and 0.8% (Cy5). (e) Patient 9 with 30% v/v DEG added to the assay. The increases were 16% (Cy3) and 15% (Cy5). (f) Detection of miR-21 and miR-7a from Patient 10 (901.67ng/µL of total RNA) showed increases of 8 % and 14% increase in Cy3 and Cy5. In all panels, yellow histograms represent the closed (quenched) biosensor state at 0 min, while green (Cy3 for miR-21) and red (Cy5 for miR-7a) histograms at 6 min reflect fluorescence increases due to miRNA recognition and biosensor activation. Note: Fluorescence intensity corresponds to biosensor opening; counts refer to the number of individual DNA origami biosensors observed.


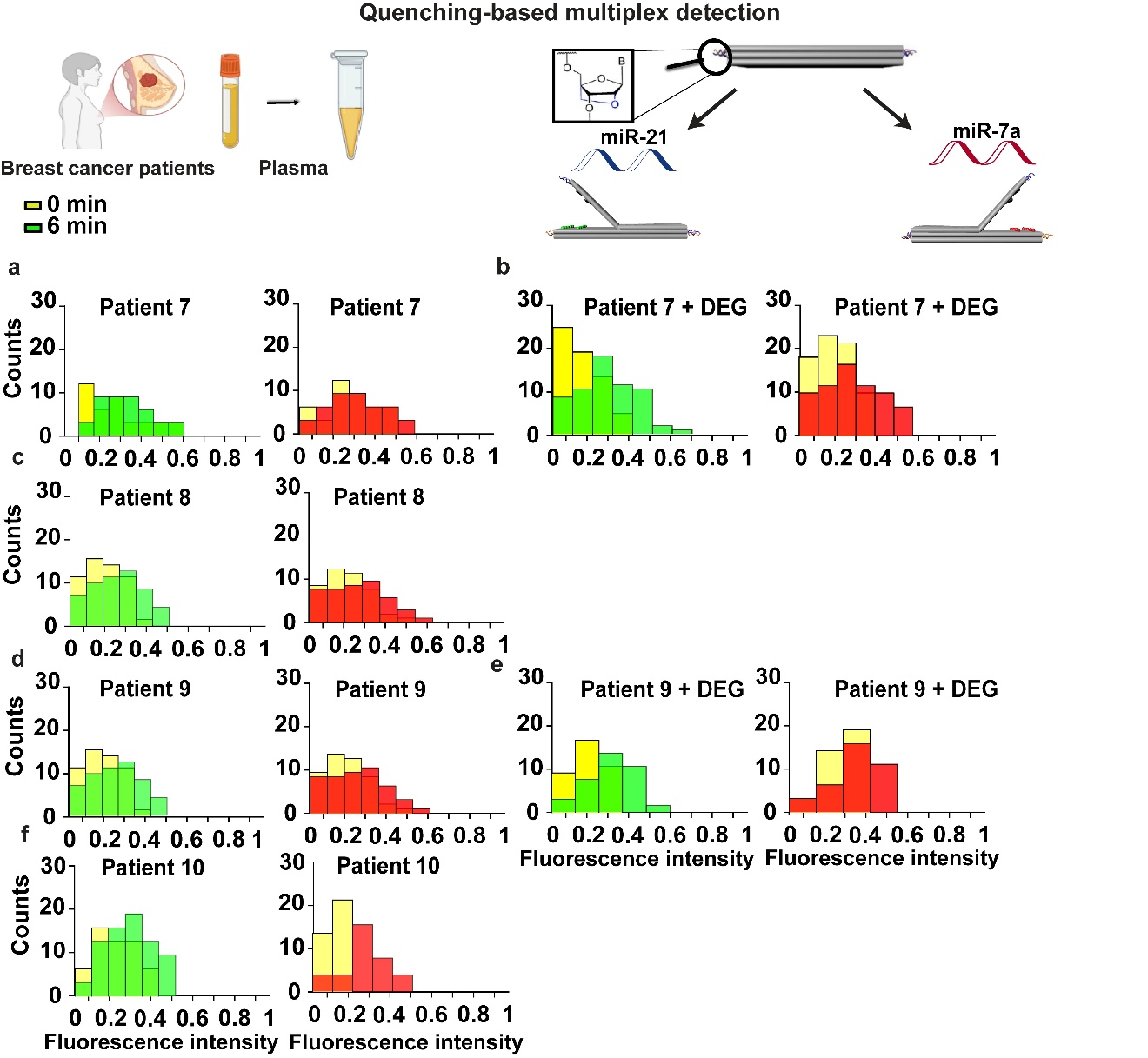


**Table S1: List of the target oligonucleotide sequences**

| **miRNAs** | **Sequence** |
| --- | --- |
| let-7a | UGA GGU AGU AGG UUG UAU AGU U |
| miR-21 | UAG CUU AUC AGA CUG AUG UUG A |
| non-target -miR-342 | UCU CAC ACA GAA AUC GCA CCC GU |
| non-target -miR-153 | UUG CAU AGU CAC AAA AGU GAU C |

**Table S2. Modified lock sequences for miR-21 detection (left side of the DNA origami biosensor).** Nucleotides that were modified are marked with an asterisk (*). All other sequences used in the biosensor design can be found in the previously published paper. ^1^

**2'-F bases within the left lock**

| 0[31] | 0[0] | AGCCCCCGATTTAGAGCTTTTGAT CAC TTT­ TGT GAC TAT G*CA A* |
| --- | --- | --- |
| 4[31] | 4[0] | CTATCGGCCTTGCTGGTAATTGAT CAC TTT TGT GAC TAT G*CA A* |
| 16[31] | 16[0] | GGTCTGAGAGACTACCTTTTTGAT CAC TTT TGT GAC TAT G*CA A* |
| 20[31] | 20[0] | CTGTCCAGACGACGACAATTTGAT CAC TTT TGT GAC TAT G*CA A* |

**LNA bases within the left lock**

| 0[31] | 0[0] | AGCCCCCGATTTAGAGCTTTTGAT CAC TTT­ TGT GAC T*A*T G*CA A |
| --- | --- | --- |
| 4[31] | 4[0] | CTATCGGCCTTGCTGGTAATTGAT CAC TTT TGT GAC T*A*T G*CA A |
| 16[31] | 16[0] | GGTCTGAGAGACTACCTTTTTGAT CAC TTT TGT GAC T*A*T G*CA A |
| 20[31] | 20[0] | CTGTCCAGACGACGACAATTTGAT CAC TTT TGT GAC T*A*T G*CA A |

| **Patient ID (used in text)** | **Sample ID** | **Group** | **Study** | **AGE** | **Primary Tumor grade** | **Primary Tumor pathology staging** | **Lymph node pathology staging** | **KI67 %** | **PR (score %)** | **ER (score %)** | **HER2 / FISH** | **Hormonal tumor subtype** | **Sample Type** |
| --- | --- | --- | --- | --- | --- | --- | --- | --- | --- | --- | --- | --- | --- |
| Patient 1 | Patient 35 | Metastatic | CSFR1 | 78 | 1 | pT2 | pN1 (1/25) N1 biii | ND | yes | yes | negative | Luminal - A | Extraction of small RNA |
| Patient 2 | Patient 32 | Metastatic | CSFR1 | 57 | 3 | cT4b | cN2a | ND | yes | yes | negative | Luminal - A | Extraction of small RNA |
| Patient 3 | A1_06 | Primary | CSFR4 | 69 | 2 | pT1a | pN0 (sn, 0/1) | 15 | 100 | 100 | negative | NA | Plasma |
| Patient 4 | A1_04 | Primary | CSFR4 | 73 | 2 | pT2 | pN1a (sn) | 25 | 95 | 2 | negative |  | Plasma |
| Pateint 5 | A1bis_08 | Primary | CSFR4 | 73 | ND | pT1c | pN0 (sn) | 20 | 100 | 0 | negative | NA | Extraction of small RNA |
| Patient 6 | A1_01 | Primary | CSFR4 | 62 | 1 | pT1b | pN1a (sn) | 5 | 95 | 70 | negative | Luminal B | Extraction of small RNA |
| Patient 7 | Patient 63 | Metastatic | CSFR1 | 52 | 3 | pT2 | 2a (7/11) | ND | no | yes | negative | Luminal - A | Plasma |
| Patient 8 | A1_01 | Primary | CSFR4 | 62 | 1 | pT1b | pN1a (sn) | 5 | 95 | 70 | negative | Luminal B | Plasma |
| Patient 9 | A1_01_2 | Primary | CSFR4 | 62 | 1 | pT1b | pN1a (sn) | 5 | 95 | 70 | negative | Luminal B | Plasma |
| Patient 10 | Patient 33 | Metastatic | CSFR1 | 66 | ND | cT4b Haut | cN1a | 10 | yes | yes | negative | Luminal - A | Plasma |

**Table S3. Mapping of patient IDs to tube labels and sample information.** For clarity and consistency, patient samples are referred to in the manuscript as “Patient 1”, “Patient 2”, etc. The corresponding original sample identifiers used during experimental processing are listed here, along with associated clinical and pathological characteristics. *Note. CSFR1, sample at the day 0 before any treatment started at the time of metastatic detection. CSFR4 -sample from time zero, before surgery of the primary tumor.*

**References**

1. Domljanovic, I. *et al.* DNA origami book biosensor for multiplex detection of cancer-associated nucleic acids. *Nanoscale* (2022) doi:10.1039/D2NR03985K.
